# Supplementary material for: How effectively can HIV phylogenies be used to measure heritability?
Source: Evol Med Public Health. 2013 Sep 13;2013(1):209–24. doi: 10.1093/emph/eot019 (PMC3850537; doi:10.1093/emph/eot019)
Supplement: Supplementary Data [file supp_2013_1_209__index.html]

How effectively can HIV phylogenies be used to measure heritability? — Supplementary Data 

# How effectively can HIV phylogenies be used to measure heritability?

## Supplementary Data

files

**Files in this Data Supplement:**

- Supplementary Data - zip file
