# Supplementary material for: How effectively can HIV phylogenies be used to measure heritability?
Source: Evol Med Public Health. 2013 Sep 13;2013(1):209–24. doi: 10.1093/emph/eot019 (PMC3850537; doi:10.1093/emph/eot019)
Supplement: Supplementary Data [file supp_eot019_suppl_data.zip › SUPPLEMENT_2.pdf]

Supplementary Information

George Shirreff[1,2], Samuel Alizon[3], Anne Cori[1], Huldrych F. Günthard[4], Oliver Laeyendecker[5,6], Ard van Sighem[7], Daniela Bezemer[7], and Christophe Fraser[1]

[1]Medical Research Council Centre for Outbreak Analysis and Modelling, Department of Infectious Disease Epidemiology, Imperial College, London, United Kingdom.
[2]Institute for Integrative Biology, ETH Zürich, Zürich, Switzerland
[3]Lab MIVEGEC UMR CNRS 5290, IRD 224, UM1, UM2, F-34394 Montpellier 5, France.
[4]Division of Infectious Diseases and Hospital Epidemiology, University Hospital of Zürich, University of Zürich, Zürich, Switzerland.
[5]National Institute of Allergy and Infectious Diseases, National Institutes of Health, Baltimore, Maryland, United States of America.
[6]Department of Medicine, Johns Hopkins University School of Medicine, Baltimore, Maryland, United States of America.
[7]Stichting HIV Monitoring, Amsterdam, Netherlands.

August 31, 2013

Contents

1 Supplementary Figures **22**

2 Supplementary Tables **28**

3 Supplementary Text **28**
3.1 Statistics for describing heritability by phylogenetic comparative analysis . . . 28
3.2 Approximate Bayesian Computation (ABC) to estimate distribution of true heritability . . . 32
3.3 Interpreting heritability . . . 33
3.4 Sample size and phylogenetic uncertainty . . . 34
3.5 Simulation method . . . 37
3.6 Accession numbers . . . 37

Bibliography **40**

List of Figures

S1 Phylogeny inferred for the *gp41* gene in the Rakai data. . . . 22
S2 Phylogeny inferred for the *p24* gene in the Rakai data. . . . 23
S3 Phylogeny inferred for all the Swiss data. . . . 24
S4 The best tree inferred from the entire Netherlands *pol* sequence dataset. . . . 25
S5 Comparison of the sensitivity of the different Hierarchical Clustering (HC) statistics to heritability on the Swiss MSM Strict phylogeny. . . . 26
S6 Comparison of the sensitivity of the different Abouheif-Moran (AM) statistics to heritability on the Swiss MSM Strict phylogeny. . . . 27
S7 Heritability estimated by ABC using the Phylogenetic Pairs method in the *p24* phylogeny of the Rakai data, using a range of different values of ϵ. . . . 33

List of Tables

S1 Characteristics of the Ugandan data. 28
S2 Characteristics of the Swiss data. 29
S3 Characteristics of the Netherlands data. 30
S4 Heritability estimates for the Swiss and Rakai under the phylogenetic mixed model. 31
S5 The effect of sample size reduction on significance in the Swiss data. 35
S6 The effect of phylogenetic uncertainty on significance in the Swiss data. 36
S7 Confidence intervals of h^2 in subtype A *p24* data from Rakai using different simulation methods. 38

1 Supplementary Figures

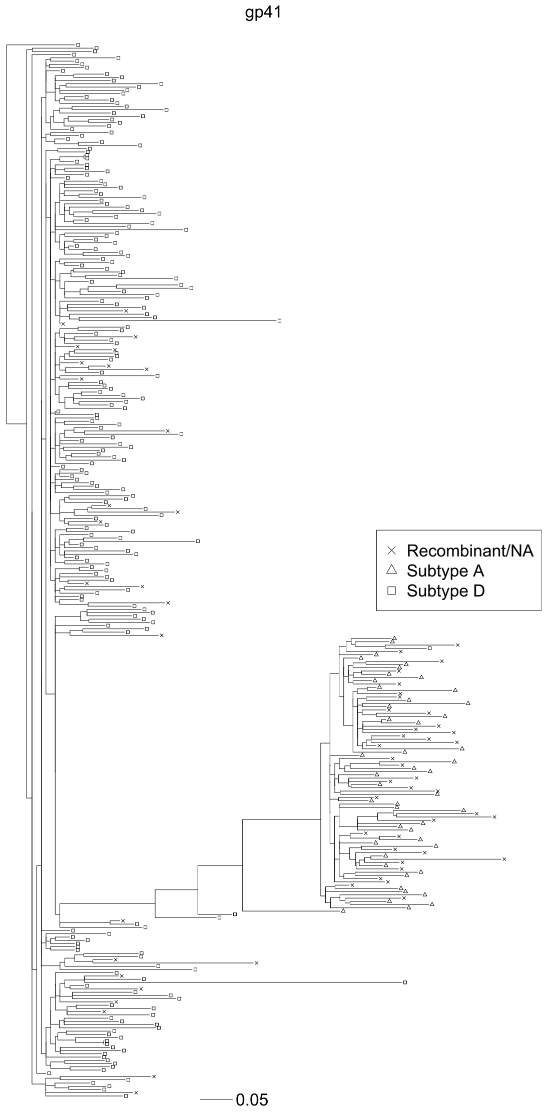

Figure S1: Phylogeny inferred for the *gp41* gene in the Rakai data.

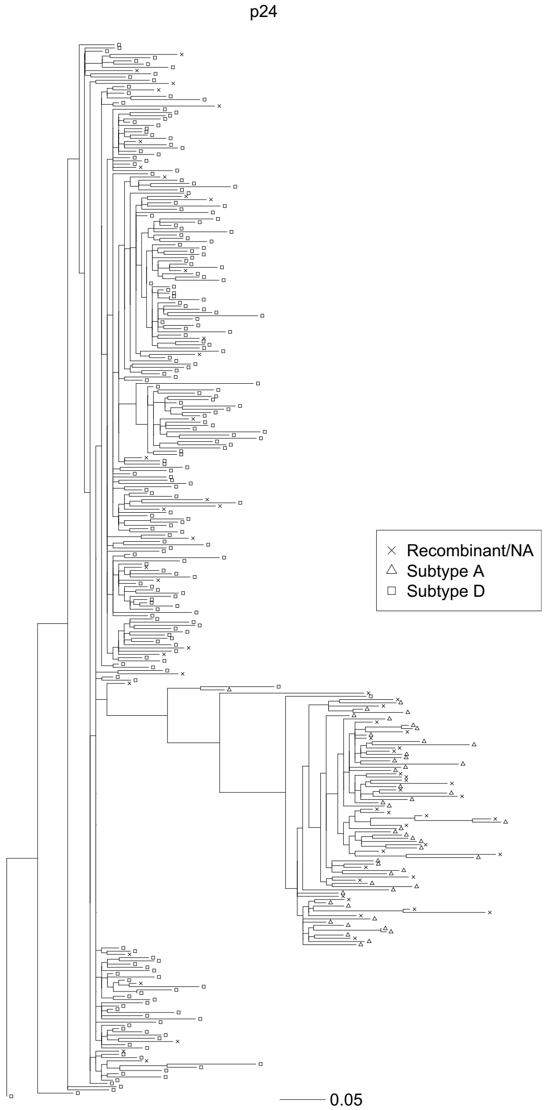

Figure S2: Phylogeny inferred for the *p24* gene in the Rakai data.

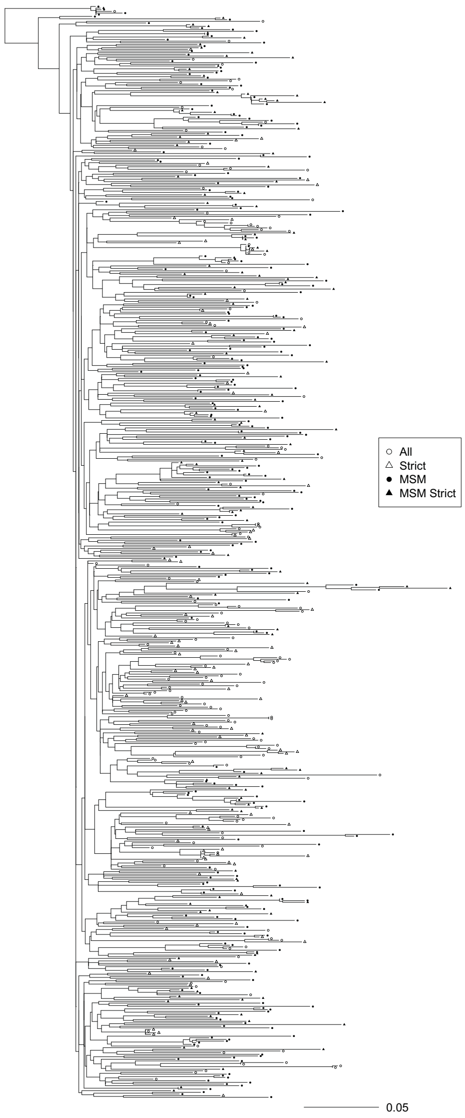

Figure S3: Phylogenetic tree for the Swiss cohort.

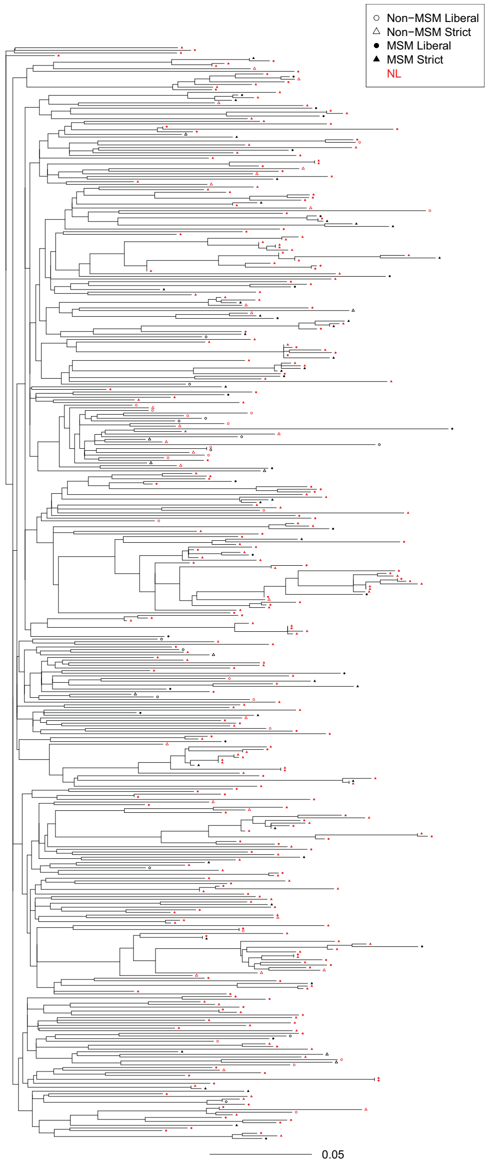

Figure S4: The best tree inferred from the entire Netherlands *pol* sequence dataset. Certain codons were excluded based on their association with drug resistance mutations. These were taken from the Stanford drug resistance database [22, 23] in 2009 as follows: in protease, amino acid positions 23, 24, 30, 32, 33, 46, 47, 48, 50, 53, 54, 76, 82, 84, 88, and 90; in reverse transcriptase, amino acid positions 41, 65, 67, 69, 70, 74, 75, 100, 101, 103, 106, 115, 151, 179, 181, 184, 188, 190, 210, 215, 230, and 236.

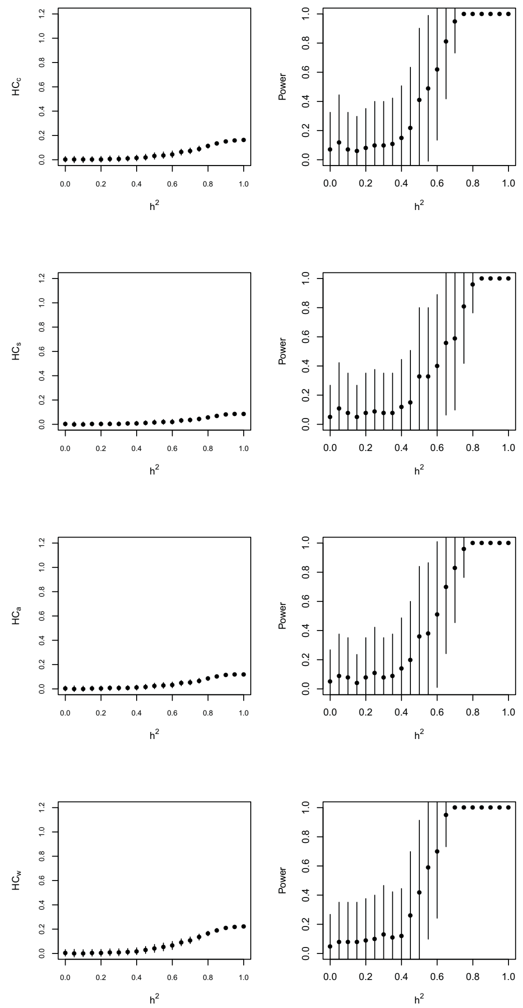

Figure S5: Comparison of the sensitivity of the different Hierarchical Clustering (HC) statistics to heritability on the Swiss MSM Strict phylogeny (‘complete’, ‘single’, ‘average’, and ‘ward’). (Left side) The relationship between heritability and the statistics under comparison. The circles and bars represent the mean and standard deviation of the sample. (Right side) The power of each statistic to detect an effect at 5% significance. The bars represent the standard deviation of the proportion.

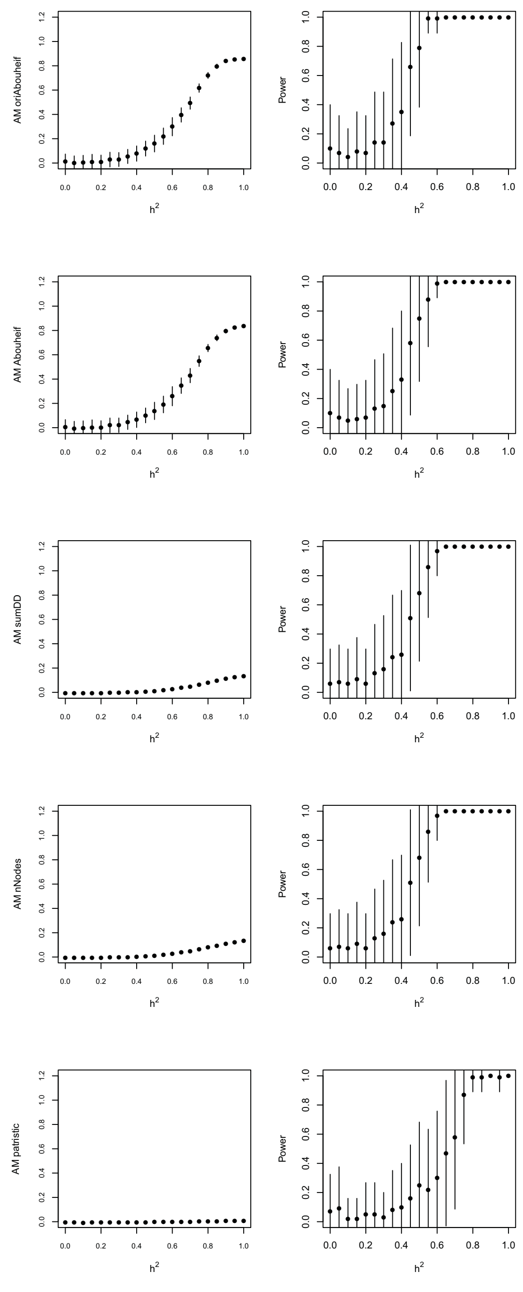

Figure S6: Comparison of the sensitivity of the different Abouheif-Moran (AM) statistics to heritability on the Swiss MSM Strict phylogeny ('oriAbouheif', 'Abouheif', 'sumDD', 'nNodes', and 'patristic'). (Left side) The relationship between heritability and the statistics under comparison. The circles and bars represent the mean and standard deviation of the sample. (Right side) The power of each statistic to detect an effect at 5% significance. The bars represent the standard deviation of the proportion.

2 Supplementary Tables

Table S1: Characteristics of the individuals from the cohort in Rakai, Uganda which are included in this study. Missing values for each variable were included as a separate category.

**Factor**	**Category**	**Number**
Locus	Either	332 (100.0%)
	gp41	325 (97.9%)
	p24	327 (98.5%)
	Both	320 (96.4%)
Sex	Male	147 (44.3%)
	Female	185 (55.7%)
Age	15-24	45 (13.6%)
	25-29	54 (16.3%)
	30-39	83 (25.0%)
	40+	34 (10.2%)
	Missing data	116 (34.9%)
Genital Ulcer Disease (GUD)	Yes	37 (11.1%)
	No	31 (9.3%)
	Missing data	264 (79.5%)
Subtype	A	48 (14.5%)
	D	221 (66.6%)
	Other/missing	63 (19.0%)

3 Supplementary Text

3.1 Statistics for describing heritability by phylogenetic comparative analysis

Phylogenetic Pairs (PP)

The newly developed PP method identifies and analyses putative transmission pairs on the phylogenetic tree. These are identified as two tips that are each others closest neighbour by branch distance. They are located by testing each tip i in turn. For each i its nearest neighbour j is located, and if i is also the nearest neighbour of j, then they are a pair. They are not required to form a monophyletic group, nor is there a maximum distance between them, since distance outliers were extremely rare.

Analysis of variance (ANOVA) is then performed as in equations 4 and 5 where Cluster indicates the pair identifier and in each case the adjusted R^2 is used as a measure of the variance explained. The difference between these two values is the additional variance explained by the model which takes into account the pairs (equation 6). This then becomes the test statistic PP (equation 7). Individuals not included in a pair are excluded.

The cofactors in addition to 'Cluster' are age, sex and genital ulcer disease (GUD) in the Rakai dataset (Table S1); age, sex and risk group in the Swiss dataset (Table S2); and age, sex, risk group, region of origin and the type of assay used to measure viral load in the Netherlands dataset (Table S3).

Table S2: Characteristics of the individuals from the Swiss HIV Cohort Study which are included in this study. Missing values for each variable were included as a separate category.

**Factor**	**Category**	**Number**
Sex	Male	554 (83.8%)
	Female	107 (16.2)
Age	15-24	53 (8.0%)
	25-29	110 (16.6%)
	30-39	280 (42.4%)
	40-49	151 (22.8%)
	50+	49 (7.4%)
	Missing data	18 (2.7%)
Risk group	Blood	2 (0.3%)
	Heterosexual	142 (21.4%)
	Injecting drug use	97 (14.7%)
	MSM	404 (61.1%)
	Other	4 (0.6%)
	Missing	12 (1.8%)
Viral load variability	Liberal only	431 (65.2%)
	Strict	230 (34.8%)

$$\text{SPVL: Cluster+\{cofactors\}} \rightarrow \text{adj}{R_c}^2 \tag{4}$$

$$\text{SPVL: \{cofactors\}} \rightarrow \text{adj}R^2 \tag{5}$$

$$\Delta(R^2) = \text{adj}{R_c}^2 - \text{adj}R^2 \tag{6}$$

$$PP = \Delta(R^2) \tag{7}$$

We ignore cofactors for the purposes of simulation, and they are not included in analyses which used simulated data. In this case the ANOVA is performed by regression of SPVL on the pair alone (equation 8), and the test statistic PP_s was the resulting adj${R_s}^2$ (equation 9).

$$\text{SPVL: Cluster} \rightarrow \text{adj}{R_s}^2 \tag{8}$$

$$PP_s = \text{adj}{R_s}^2 \tag{9}$$

Hierarchical Clustering (HC)

The newly developed HC method is similar to the PP method but it combines individuals into clusters of two or more according to a threshold phylogenetic distance. Short distances give small clusters and many

Table S3: Characteristics of the individuals from the Netherlands which are included in this study. Other characteristics of these individuals which were also used in this analysis are displayed in Table S3. The following categories of region of origin were combined as cofactors: Netherlands & Western Europe; Latin America & Caribbean; Eastern & Central Europe; South East Asia & Oceania. The viral load assay categories were NASBA (Nucleic Acid-Sequence Based Amplification), RT-PCR (Reverse Transcriptase Polymerase Chain Reaction) and bDNA (branched DNA signal amplification). The type of assay used may influence the viral load measurements. When analyzing the Netherlands data with the PP method we were able to control for the type of viral load assay used [10]. This factor was previously found to be significant in the measurement of SPVL [10]. Since these data were not available for the Swiss data, it is possible that the analysis of those datasets is somewhat confounded by the choice of assay. In the Rakai data, the same type of assay was always used. Missing values for each variable were included as a separate category.

**Factor**	**Category**	**Number**
Sex	Male	394 (95%)
	Female	22 (5%)
Region of origin	Netherlands	332 (80%)
	Western Europe	30 (7%)
	Latin America	12 (3%)
	Central Europe	7 (2%)
	Caribbean	7 (2%)
	South East Asia	5 (1%)
	Sub-Saharan Africa	5 (1%)
	Eastern Europe	4 (1%)
	Oceania	1 (0.2%)
	Unknown	13 (3%)
Risk category	MSM	348 (84%)
	IDU	11 (3%)
	HET	33 (8%)
	Blood transfusion	1 (0.2%)
	Unknown	23 (6%)
Age at seroconversion	15-24	34 (8%)
	25-29	85 (20%)
	30-39	170 (41%)
	40-49	96 (23%)
	50+	31 (7%)
Type of viral load assay	NASBA	113 (27%)
	RT-PCR	159 (38%)
	bDNA	134 (32%)
	Unknown	10 (2%)
Strict	Yes	246 (59%)
	No	170 (41%)

individuals are not included in a cluster, while larger distances give larger clusters and more inclusion. There is no intuitive ideal cluster size, proportion included or number of clusters to use, so the method integrates over the range of clustering distances. There are also 4 possible clustering algorithms, implemented using the *hclust* method in R [24], although the 'ward' algorithm has been used as the default for this analysis.

1. 'complete': joins an individual to a cluster if it is linked to (within the threshold distance of) every other member of the cluster.

2. 'single': joins an individual to a cluster if it is linked to any other member of the cluster.

Table S4: Heritability values of the Swiss and Rakai data under the phylogenetic mixed model [13]. They are described as $^*h^2$ to differentiate them from our values estimated by simulation.

**Cohort**	**Subdivision**	$^*h^2$
Swiss	All Liberal	0.06
	Strict	0.15
	MSM	0.01
	MSM Strict	0.12
Rakai	Subtype A *gp41*	0.00
	Subtype A *p24*	0.00
	Subtype D *gp41*	0.14
	Subtype D *p24*	0.00

3. 'average': joins an individual to a cluster if the average distance to every other member of the cluster is lower than the threshold.
4. 'ward' (used by default): calculates the least squares cost of each cluster, based on the distances between its members. If the cost of joining two clusters is less than the threshold then they will be combined.

Individuals are assigned to clusters at a given threshold distance d. A pair of ANOVAs is then performed exactly as in equations 4 and 5, and the additional variance explained by clustering at this distance is also calculated as in equation 6.

The test statistic, HC, is then calculated by summing over all possible threshold distances (equation 10), and therefore measures the explanatory power of clustering at all depths of the tree.

$$HC = \Delta d \sum_{d} \left(\Delta(R^2)(d) \right) \tag{10}$$

As with the PP method, cofactors are only included in the randomisation test, so when simulations are involved a single ANOVA is done for each clustering distance, as in equation 8.

Mantel test

The Mantel test measures correlation between two distance matrices [16], in this case the phylogenetic distance matrix, and the SPVL trait difference matrix. The statistic is the sum of the product of each cell and the corresponding cell in the other matrix (excluding the diagonal). The method was implemented in R [24] using the *ade4* package [5].

Blomberg's K

In this method the calculation of the value K is based on independent contrasts of the trait values in the phylogeny. Pairs of sister tips (a and b) are contrasted independently [7, 2] by dividing the squared difference in their trait values (T) by the branch length (d) separating them, and taking the square root (equation 11).

$$\text{Contrast}_{ab} = \sqrt{\frac{(T_a - T_b)^2}{d_{ab}}} \tag{11}$$

These tips are then removed, and their parent node, c, ascribed a trait value from an average of the value at each daughter tip, weighted by the reciprocal of the branch length from the parent node to each tip 12.

$$T_c = \frac{T_a \frac{1}{d_{ac}} + T_b \frac{1}{d_{bc}}}{\frac{1}{d_{ac}} + \frac{1}{d_{bc}}} \tag{12}$$

This new tip can then be compared with its sister tip, and this continues until every pair has been contrasted, and the variance is then calculated. The variance of these Phylogenetic Independent Contrasts (PICv) is high (low) when phylogenetic signal is low (high).

The PICv is dependent on the size and shape of the tree, so cannot be directly compared between phylogenies. The K statistic, however, can be. This is calculated by dividing the variance in the tip data by the value of PICv, giving a number which is high in the presence of phylogenetic signal. K is then calculated by dividing this value by its expected value under a Brownian motion model of evolution. If K=0, this indicates no phylogenetic signal, while K=1 implies evolution according to Brownian motion. K can also exceed one, for example if the trait is selected in different directions on different branches of the tree. This statistic was measured in R [24] using the phylosignal function from the package *picante* [15].

Pagel's λ

The shared evolutionary history of the tips on a phylogeny can be expressed as a variance-covariance matrix of size $n \times n$ where n is the number of tips. The values along the diagonal represent the distance between each tip and the root, and the off-diagonal values give the distance between the root and the most recent common ancestor of the two tips. This value therefore captures the shared evolutionary history between these two tips, with higher values representing a greater amount of shared history, and a consequent similarity in trait values.

These covariances can be used to simulate these trait values, which can be fit to the true data. The λ statistic is a multiplier which transforms the covariances to maximise the fit between the simulated and the real tip data. If λ=0, the phylogeny has no effect . If λ=1, trait evolution can be best described by a Brownian motion model, and $\lambda > 1$ is also possible [9]. It was implemented in R [24] using the *phylosig* function [18, 21].

Abouheif-Moran test

The Abouheif-Moran (AM) statistic is the sum of the differences between the trait values for each pair of tips, weighted by the reciprocal of the distance between them. There are five different methods for measuring the distance between tips, although number two, 'Abouheif', has been used as the default for this analysis. It was implemented in R [24] using the *adephylo* package [14].

1. 'oriAbouheif': derived from the test for serial independence [1], this measure is the inverse of the product of the number of branches descending from each node in the path connecting the two tips [19].
2. 'Abouheif' (used by default): as above but the diagonals in the proximity matrix are set to zero [19].
3. 'sumDD': as in the "Abouheif" method, but it measures the inverse of the sum of the number of descending branches, rather than the inverse of the product.
4. 'nNodes': The inverse of the number of nodes on the path connecting the two tips.
5. 'patristic': The inverse of the branch length on the path separating two tips.

3.2 Approximate Bayesian Computation (ABC) to estimate distribution of true heritability

ABC is used to identify a distribution of an unknown parameter when its likelihood cannot be calculated [4]. The parameter of interest, in this case h^2, is drawn from a prior distribution, in this case uniform between 0 and 1. Data are simulated as above, and the phylogenetic comparative statistic is calculated Z. If it is

sufficiently close to the true statistic, $\hat{Z}$, then the corresponding value of h^2 is included in the estimated posterior distribution, and this is repeated until 1000 posterior estimates are obtained. The required closeness to the true statistic is determined by a tolerance parameter ϵ. If the inequality 13 is satisfied, that value of h^2 is accepted.

$$\hat{Z} - \epsilon < Z(h^2) < \hat{Z} + \epsilon \tag{13}$$

In order to pick an appropriate value of ϵ, we performed some preliminary work, in which values of h^2 between 0 and 1 in increments of 0.1 were each used to simulate 100 datasets. For each value of h^2, the standard deviation of the statistic in question was calculated. The median standard deviation across all values of h^2 then became a measure of the expected spread of this statistic. The value of ϵ was then set at 10% of this medium, based on the performance of such a ratio in preliminary studies (data not shown). We subsequently validated the level of ϵ by comparing the resulting posterior distribution of h^2 across a range of values of ϵ (Figure S7). Although we display only a single subdivision, it is representative of the robustness of using the value of ϵ as specified in this paragraph. The shape of the distribution does not become more refined above a ratio of 10, although the computational intensity increases rapidly, because the smaller size of the tolerated distribution requires more trials to find 1000 successful ones.

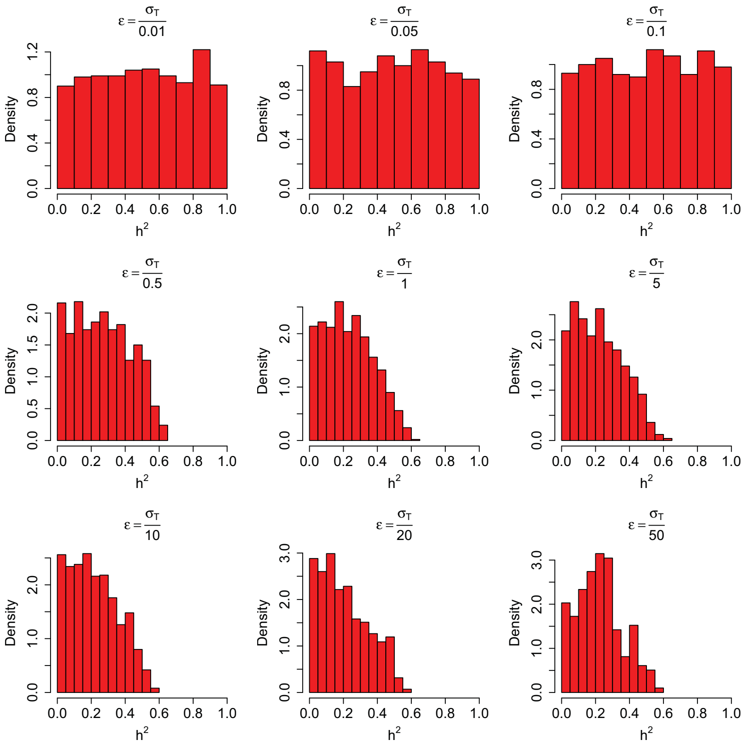

Figure S7: Heritability estimated by the Phylogenetic Pairs method in the *p24* phylogeny of the Rakai data, using a range of different values of ϵ.The value of ϵ is written above the plot, in which σ_T is the median standard deviation in the statistic, as estimated in preliminary work (data not shown).

3.3 Interpreting heritability

Heritability is measured by comparing trait values between generations of the organism, and underlies the response of the trait to natural or artificial selection. In the context of communicable diseases, we can consider the host infection to be the "organism", or unit of selection, the characteristics of the infection to

be the trait, and heritability is measured by comparing these characteristics between one infection and the next.

Both Blomberg's K and Pagel's λ have been linked directly with h^2, but they can both exceed one [9, 3] which h^2 can not since it is a proportion. The PP statistic also represents the quantity of variation in SPVL which can be explained by the viral genotype (via the phenotype of a person carrying genetically similar virus), and it may therefore be linked directly to h^2. However, it is not clear that how these methods scale with h^2. The other methods (HC, Mantel and AM) also provide statistics which are not on the scale of h^2.

Simulations in previous work have shown that an input variable closely related to h^2 is strongly correlated with Blomberg's K and Pagel's λ [2] but in neither case is the relationship linear. In this study, we use simulation to link these values directly with heritability, h^2 as defined in equation 1. Equation 2 represents the relationship between a given donor and recipient. This can be written instead as a regression equation, 14, where $y = V_D$ (the daughter node, or recipient in a transmission pair), $x = V_P$ (the parent node, or donor in a transmission pair).

$$y = a + bx + \xi \tag{14}$$

We set $a = \bar{\mu}\sqrt{1-(h^2)^2}$ (the intercept) which is the mean $\log_{10}$ SPVL of a recipient infected by a donor of $\log_{10}$ SPVL=0. The value $\xi = (M - \bar{\mu})\sqrt{1-(h^2)^2}$ represents random noise in the relationship, and is normally distributed around zero (see equation 3). Under these assumptions the value b, which is the regression slope between the donor and recipient, is equal to h^2. This result has been shown previously by Falconer[6].

In addition, the preservation of variance in trait values between donors and recipients in the simulation equation implies that the value h^2 is also equivalent to the correlation coefficient between a donor and a recipient, which is the regression slope scaled for the respective variances of the two variables.

Note that studies which measure heritability between couples deal with direct descent between adjacent generations. Lack of complete sampling in the datasets we use to build phylogenies precludes such direct descent and instead focusses on correlation between individuals more than one generation apart, so the methods we use naturally measure weaker heritability than studies of transmission pairs.

3.4 Sample size and phylogenetic uncertainty

We tested the possibility that high significance in the smaller datasets of the Swiss data is simply an artifact of a smaller tree, even if smaller trees do not have greater phylogenetic signal. We artificially created 100 datasets of the size of the MSM Strict subdivision (n=134) by randomly sampling without replacement from the entire dataset (n=661). The randomization test was then performed on each sample and the mean was compared with the results from the true All and MSM Strict subdivisions (Table S5). Significant heritability was much less likely to be found in the randomly smaller subdivision than in the MSM Strict subdivision. This indicates that the strength of significance in our results does not simply reflect sample size, but rather some effect of the patient inclusion criteria.

Throughout this work the phylogenetic trees have been assumed to be correct. This is unlikely, due to sequencing errors, phylogenetic inference, and incomplete and inhomogeneous sampling. We address this by validating the randomisation test analysis of the MSM Strict subdivision of the Swiss data using deliberately perturbed trees, in the manner described by Alizon et al. [2]. The data are perturbed 100 times by randomly reassigning a proportion (3%, 10% or 20%) of the tips, and the randomisation test subsequently performed (Table S6). As expected, the significance of the test decreases as the tree becomes more uncertain, but a perturbation of 3% is tolerated.

Table S5: The effect of sample size reduction on significance in the Swiss data. This was examined by randomly resampling from the All Liberal dataset (n=661) a sample of the size of the MSM Strict dataset (n=134). Borderline significance (p<0.1) is in blue, and formal significance (p<0.05) is in red.

	All (n=661)		All resampled (n=134)		MSM Strict (n=134)	
	Z	p	Z*	p*	Z	p
PP	-0.0892	0.817	-0.0291	0.529	0.5948	0.005
HC complete	0.0082	0.214	-0.005	0.544	0.0494	0.032
HC single	0.0056	0.159	-0.0025	0.527	0.0343	0.040
HC average	0.0041	0.315	-0.0037	0.538	0.0462	0.024
HC ward	0.0077	0.315	-0.0059	0.544	0.0634	0.023
Mantel	-0.014	0.712	0.0052	0.476	0.0499	0.207
Blomberg K	0.0023	0.305	0.3334	0.514	0.5933	0.080
PICv	1594	0.307	331	0.472	5.579	0.002
Pagel λ	0.1048	0.016	0.0666	0.512	0.6459	0.016
AM oriAbouheif	-0.0126	0.680	-0.0052	0.525	0.1273	0.023
AM Abouheif	-0.0138	0.671	-0.0133	0.528	0.1256	0.019
AM sumDD	0	0.166	-0.0086	0.525	0.0133	0.016
AM nNodes	0	0.166	-0.0086	0.525	0.0133	0.016
AM patristic	0.0018	0.320	-0.007	0.545	0.0002	0.054

PP – Phylogenetic Pairs
HC – Hierarchical Clustering
PICv – Variance of Phylogenetic Independent Contrasts
AM – Abouheif Moran tests
*average over 100 resamplings

Table S6: The effect of phylogenetic uncertainty on significance in the Swiss MSM Strict subdivision, by random replacement of 3, 10 or 20% of tips. Borderline significance ($p<0.1$) is in blue, and formal significance ($p<0.05$) is in red.

	MSM Strict (n=134)		Perturb 3% (n=134)		Perturb 10% (n=134)		Perturb 20% (n=134)	
	Z	p	Z*	p*	Z*	p*	Z*	p*
PP	0.5948	0.005	0.5642	0.009	0.4808	0.030	0.3745	0.086
HC complete	0.0494	0.032	0.0472	0.047	0.0428	0.079	0.035	0.137
HC single	0.0343	0.040	0.0328	0.061	0.0304	0.093	0.0248	0.151
HC average	0.0462	0.024	0.0441	0.039	0.0401	0.068	0.0328	0.122
HC ward	0.0634	0.023	0.0604	0.033	0.0544	0.061	0.0444	0.117
Mantel	0.0499	0.207	0.0485	0.221	0.0431	0.253	0.0449	0.259
Blomberg K	0.5933	0.080	0.5819	0.092	0.5541	0.136	0.5203	0.202
PICv	5.579	0.002	5.848	0.018	6.09	0.047	6.636	0.104
Pagel λ	0.6459	0.016	0.5858	0.040	0.442	0.125	0.2609	0.303
AM oriAbouheif	0.1273	0.023	0.121	0.033	0.1067	0.067	0.0793	0.146
AM Abouheif	0.1256	0.019	0.119	0.028	0.1045	0.058	0.0771	0.130
AM sumDD	0.0133	0.016	0.0123	0.025	0.01	0.053	0.0057	0.129
AM nNodes	0.0133	0.016	0.0123	0.025	0.01	0.053	0.0057	0.129
AM patristic	0.0002	0.054	-0.0003	0.073	-0.0011	0.100	-0.002	0.150

PP – Phylogenetic Pairs
HC – Hierarchical Clustering
PICv – Variance of Phylogenetic Independent Contrasts
AM – Abouheif Moran tests
*average over 100 resamplings

3.5 Simulation method

Previous work has identified a relationship between transmission fitness and SPVL [8], which suggests transmitting nodes on the tree are likely to have SPVL values closer to the optimum for transmission fitness, which we ignore. We disregard a general, but non-significant upward trend in virulence [11], which was found to be significant in the Netherlands [10] if not Switzerland [17]. However, we assume that this does not lead to substantial differences in estimates of phylogenetic signal.

The simulation method was chosen for its parsimony and is based on a simplistic and directionless model of evolution which maintains the population variance of the trait. Results in the main text were based on the assumption that a single branch segment on the tree represents a single transmission, and that mutation of the viral genotype with respect to virulence is the same, regardless of the branch length. We note that this probably underestimates the number of generations separating two tips, leading to underestimates of heritability, especially at low coverage.

We address this problem with an alternative method of simulation, which allows more generations to occur on longer branches (Table S7), but this requires estimates for the between-host generation time i.e. waiting time between transmission events, and the tree clock rate, both of which are uncertain (the tip dates explained only 16% of the genetic divergence in the Rakai data).

The number of generations between each parent and daughter node (P and D respectively) is represented by g_{PD} (equation 15). At least one generation is assumed, but the number in excess of this is taken from a Poisson distribution where l_{PD} is the distance between the two nodes in mean substitutions per site, r is the rate of evolution on the phylogeny in substitutions per site per year, and T_g is the mean generation time of HIV-1.

$$g_{PD} = 1 + \text{Poisson}\left(\frac{l_{PD}}{rT_g}\right) \tag{15}$$

The SPVL at each daughter node is then given as in equation 2 but allowing for more than one generation. The random component, M_b, represents the mean of g_{PD} normal distributions M from distribution 3, and therefore has the same mean, but a variance reduced by a factor g_{PD} (equations 16 and 17).

$$V_D = (h^2)^{g_{PD}} V_P + M_b \left(\sum_{i=0}^{g_{PD}-1} (h^2)^i\right) \sqrt{1-(h^2)^2} \tag{16}$$

$$M_b \sim N(\bar{\mu}, \frac{{\sigma_P}^2}{g_{PD}}) \tag{17}$$

The mean generation time in the Rakai dataset was measured as 4.3 years [12]. We applied it only to the *p24* gene in the subtype A infected individuals, because this subdivision was the most clock-like. The substitution rate, 8.8×10^{-3} per site per year, explained 16% of the divergence from the founder, as estimated using Path-O-Gen [20]. The resulting heritability is compared with the heritability from the original method of simulation in Table S7. The alternative method found much higher estimates of h^2, but the confidence intervals were wider than for the original method.

3.6 Accession numbers

Rakai: gp41 GQ253897, GQ333484.1, GQ253816.1, GQ333668.1, GQ333677, GQ333953.1, GQ334139.1, GQ334075.1, GQ253844.1, GQ333816.1, GQ333489.1, GQ334160.1, GQ333697.1, GQ333747.1, GQ333773.1, GU046794, GQ253845.1, GQ253847.1, GQ333959.1, GQ333805.1, GQ253855.1, GQ333764.1, GQ334038.1, GQ333721.1, GQ253858.1, GQ334091.1, GQ334034.1, GQ333574.1, GQ253885.1, GQ334006.1, GQ333884.1, GQ334143.1, GQ333949.1, GQ333984.1, GQ253831.1, GQ334171.1, GQ253802.1, GQ333693.1, GQ334068.1,

Table S7: Confidence intervals of h^2 in subtype A *p24* data from Rakai using the original simulation method and an alternative which allows for multiple transmissions to have occurred between nodes on the phylogeny. Intervals in which the lower bound is above zero are shown in red.

	Subtype A *p24 (n=47)*	Original		Alternative	
	Z	h^2	95% CI	h^2	95% CI
PP_s	-0.0887	0.02	(0.00,0.81)	0.42	(0.00,1.00)
HC_s ward	-0.0044	0.08	(0.00,0.73)	0.32	(0.00,0.94)
Mantel	0.0243	0.39	(0.00,0.59)	0.83	(0.00,1.00)
Blomberg K	0.4516	0.43	(0.00,0.58)	0.88	(0.00,1.00)
Pagel λ	0.0001	0.10	(0.00,0.54)	0.09	(0.00,0.93)
AM Abouheif	-0.0406	0.08	(0.00,0.56)	0.16	(0.00,0.76)

PP_s – Phylogenetic Pairs by simulation
HC_s – Hierarchical Clustering by simulation
PICv – Variance of Phylogenetic Independent Contrasts
AM – Abouheif Moran tests

GQ333852.1, GQ333834.1, GU017772.1, GU017778.1, GU017779.1, GU017786.1, GQ333988.1, GQ334061.1, GQ253795.1, GQ333652.1, GQ333624.1, GQ253854.1, GQ253818.1, GQ333735.1, GQ253806.1, GQ334032.1, GQ333894.1, GQ333643.1, GQ334113.1, JQ405214, GQ333812.1, GQ333683.1, GQ333967.1, GQ334035.1, GQ333678.1, GQ253828.1, GQ333695.1, GQ334062.1, GQ333720.1, GQ334078.1, GQ333751.1, GQ253850.1, GQ333920.1, GQ333785.1, GQ333673.1, GQ333465.1, GQ333973.1, GQ253869.1, GQ253851.1, GQ333487.1, GQ333927.1, GQ333531.1, GQ333941, GQ333800.1, GQ334105.1, GQ333626.1, GQ334161.1, GQ333522.1, GU046793, GQ253883.1, GQ334182, GQ253865.1, GQ334121.1, GQ334174.1, GQ333506.1, GQ334074.1, GQ333494.1, GQ333776.1, GQ253857.1, GQ253799.1, GQ253861.1, GQ333549.1, GQ334159.1, GQ334057.1, GQ333771.1, GQ333548.1, GQ253801.1, GQ333529.1, GQ253881.1, GQ253886.1, GQ334101.1, GQ253833.1, GQ333756.1, GQ333981.1, GQ334132.1, GQ253860.1, GQ333709.1, GQ333710.1, GQ333490.1, GQ253820.1, GQ334049.1, GQ333923.1, GQ333758.1, GQ333564.1, GQ333847.1, GQ334008.1, GQ333477.1, GQ333696.1, GQ253895.1, GQ334043.1, GQ333675.1, GQ253856.1, GQ253882.1, GQ333536.1, GQ253804.1, GQ333835.1, GQ253879.1, GQ333665.1, GQ333746, GQ253824.1, GQ333901.1, GQ253896.1, GQ333525.1, GQ333528.1, GQ333596.1, GQ333974.1, GQ333485.1, GQ333692.1, GQ253796.1, GQ333767.1, GQ333804.1, GQ333741.1, GQ253811.1, GQ333615.1, GQ334123.1, GQ333551.1, GQ253889, GQ333737.1, GQ253877.1, GQ333934.1, GQ333900.1, GQ333778.1, GQ334017.1, GQ333936.1, GQ333727.1, GQ253808.1, GQ333622.1, GQ333723.1, GQ334109.1, GQ253880.1, GQ333926.1, GQ333598.1, GQ253826.1, GQ253809.1, GQ333595.1, GQ253873.1, GQ253853.1, GQ333556, GQ333582.1, GQ333488.1, GQ253884.1, GQ333777.1, GQ333821.1, GQ333534.1, GQ253894.1, GQ253846.1, GQ253810.1, GQ333641.1, GQ253827.1, GQ334129.1, GQ333633.1, GQ333547.1, GQ333738.1, GQ253862.1, GQ333947.1, GQ333869.1, GQ334089.1, GQ253825.1, GQ334016.1, GQ333557.1, GQ333880.1, GQ333558.1, GQ333718.1, GQ253870.1, GQ333500.1, GQ333681.1, GQ334108, GQ253803.1, GQ334102.1, GQ253829.1, GQ253863.1, GQ333661.1, GQ334157.1, GQ253887.1, GQ253849.1, GQ333867.1, GQ333690.1, GQ333486.1, GQ253817.1, GQ253791.1, GQ253834.1, GQ333569.1, GQ333760.1, GQ253892.1, GQ333694.1, GQ253876.1, GQ333952.1, GQ333700.1, GQ333855.1, GQ253805.1, GQ334053.1, GQ333982.1, GQ334142.1, GQ334176.1, GQ333997.1, GQ333575.1, GQ333806.1, GQ333542.1, GQ333698.1, GQ333565.1, GQ253848.1, GQ253842.1, GQ333611.1, GQ334021.1, GQ333601, GQ333929.1, GQ333635.1, GQ333478.1, GQ333502.1, GQ333989.1, GQ334124.1, GQ333940, GQ333704.1, GQ334175, GQ253822.1, GQ253821.1, GQ333523.1, GQ253890.1, JQ405213, GQ253794.1, GQ333845.1, GQ333983.1, GQ253807.1, GQ333510.1, GQ333656.1, GQ333475.1, GQ253852.1, GQ334106.1, GQ334097.1, GQ334026.1, GQ333794.1, GQ334119.1, GQ333463.1, GQ333905.1, GQ253792.1, GQ253836.1, GQ253797.1, GQ253838.1, GQ334112.1, GQ253840.1, GQ333793.1, GQ253875.1, GQ333942.1, GQ333957.1, GQ334115.1, GQ333921.1, GQ333600.1, GQ253888.1, GQ253866.1, GQ253841.1, GQ333539.1, GQ253832.1, GQ253878.1, GQ333520.1, GQ333819.1, GQ253837.1, GQ333985.1, GQ333669, GQ333640.1, GQ253830.1, GQ333511.1, GQ333882.1, GQ333979.1, GQ333728.1, GQ333912.1, GQ333896.1, GQ333898.1, GQ253891, GQ333903.1, GQ333889.1, GQ333932.1, GQ253874.1, GQ253871.1, GQ333638.1, GQ334079.1, GQ333526.1, GQ253823.1, GQ253864.1, GQ253814.1, GQ333732.1, GQ253793.1, GQ333790.1, GQ333755.1, GQ333632.1, GQ334181, GQ334003.1. **Rakai: p24** GQ253725,

GQ253667, GQ253701, GQ333179, GQ253694, GQ253668, GQ333010, GQ333390, GQ332988, GQ332778, GQ333198, GQ332818, GQ332796, GQ333248, GQ333255, GQ332938, GQ253673, GQ332928, GQ253711, GQ333345, GQ333201, GQ253721, GQ253781, GQ253710, GQ332951, GQ332849, GQ253784, GQ332848, GQ333440, GQ253777, GQ333414, GQ253681, GQ333015, GQ333223, GQ333024, GQ333304, GQ333426, GQ253716, GQ253695, GQ253706, GQ253744, GQ333361, GQ333088, GQ332879, GQ253754, GQ333235, GQ253780, GQ332963, GQ333319, GQ332985, GQ332786, GU046804, GQ253705, GQ253731, GQ333360, GU017752, GQ333259, GQ332866, GQ333389, GQ253786, GQ333094, GQ333194, GQ333357, GQ332971, GQ332815, GQ333086, GQ333211, GQ333291, GQ333318, GQ332910, GQ332799, GQ333043, GQ253755, GQ332955, GQ253776, GQ333148, GQ333417, GQ333053, GQ333341, GQ333439, GQ253759, GQ333035, GQ332843, GQ333059, GQ332790, GQ332770, GQ333239, GU017748, GQ332869, GQ332859, GQ332858, GQ332789, GQ332830, GQ253730, GQ253771, GQ332982, GQ332777, GQ333382, GQ333293, GQ332992, GQ332829, GQ333329, GQ253774, GQ253680, GQ253736, GQ333395, GQ333218, GQ333040, GQ333139, GQ332819, GQ333042, GQ253788, GQ333214, GQ253775, GQ332997, GQ332998, GQ333334, GQ333387, GQ332986, GQ333403, GQ253789, GQ333452, GQ253677, GQ333222, GQ332810, GQ333007, GQ333461, GQ333186, GQ253697, GQ332787, GQ333409, GQ332897, GQ253724, GQ253728, GQ333317, GQ332776, GQ332788, GQ333347, GQ253764, GQ333270, GQ333166, GQ253679, GQ332929, GQ333338, GQ253714, GQ253729, GQ333076, GQ333236, GQ333231, GQ333072, GQ333397, GQ333454, GQ332844, GQ333207, GQ333358, GQ253783, GQ253752, GQ253726, GQ253718, GQ253700, GQ253737, GQ333104, GQ253741, GQ332768, GQ253769, GQ333351, GQ253684, GQ333061, GU046802, GQ333373, GQ253703, GQ253702, GQ253758, GQ332823, GQ333130, GQ332865, GQ333384, GQ333137, GQ333449, GQ253778, GQ253720, GQ333273, GQ333230, GQ332962, GQ253678, GQ253713, GQ333142, GQ332883, GQ332965, GQ333388, GQ332783, GQ332878, GQ253672, GQ253709, GQ333271, GQ253735, GQ333460, GQ253733, GU017754, GQ333310, GQ332917, GQ332908, GQ332801, GQ253790, GQ253717, GQ333406, GQ333028, GQ253699, GQ333080, GQ333089, GQ333060, GQ333048, GQ332983, GQ333269, GQ332957, GQ333019, GQ333175, GQ333241, GQ253773, GQ332882, GQ332980, GQ332862, GQ253685, GQ253756, GQ333016, GQ253760, GQ333156, GQ253669, GQ253671, GQ332969, GQ332842, GQ333183, GQ332904, GQ333324, GQ253698, GQ333422, GQ333004, GQ333039, GQ332834, GQ333380, GQ253687, GQ332841, GQ253676, GQ253762, GQ253757, GQ333192, GQ253734, GQ333212, GQ332816, GQ333177, GQ333263, GQ253732, GQ333392, GQ333401, GQ333090, JQ405215, GQ333314, GQ333022, GQ253682, GQ253670, GQ253722, GQ253686, GQ332924, GQ332794, GQ332802, GQ333097, GQ333193, GQ333008, GU017761, GQ333025, GQ333300, GQ333033, GQ333101, GQ333144, GQ332922, GQ253785, GQ333372, GQ332785, GQ333116, GQ333032, GQ332919, GQ333226, GQ253767, GQ333453, GQ253766, GQ333441, GQ333294, GQ333216, GQ253674, GQ332943, GQ333435, GQ332995, GQ332894, GQ333260, GU017756, GQ253704, GQ333425, GQ253727, GQ253763, GQ332813, GQ333274, GQ253666, GQ253787, GQ333221, GQ332987, GQ333056, GQ333190, GU017759, GQ333301, GQ333284, GQ332836, GQ253691, GQ333268, GQ333165, GQ332811, GQ333046, GQ253683, GQ332978, GQ253779, GQ333219, GQ333246, GQ253782, GQ332881, GQ253768, GQ253770, GQ332925, GQ332916.

The data from Switzerland and the Netherlands are available by application to the Swiss HIV Cohort Studies (shcs.ch) and Stichting HIV Monitoring (hiv-monitoring.nl), respectively. More information is available from the authors.

References

[1] E. Abouheif. “A method for testing the assumption of phylogenetic independence in comparative data”. In: *Evolutionary Ecology Research* 1.8 (1999), pp. 895–909.

[2] S. Alizon et al. “Phylogenetic Approach Reveals That Virus Genotype Largely Determines HIV Set-Point Viral Load”. In: *Plos Pathogens* 6.9 (2010), e1001123.

[3] S. P. Blomberg, T. Garland, and A. R. Ives. “Testing for phylogenetic signal in comparative data: Behavioral traits are more labile”. In: *Evolution* 57.4 (2003), pp. 717–745.

[4] K. Csillery et al. “Approximate Bayesian Computation (ABC) in practice”. In: *Trends in ecology and evolution* 25.7 (2010), pp. 410–8.

[5] S. Dray and A. B. Dufour. “The ade4 package: Implementing the duality diagram for ecologists”. In: *Journal of Statistical Software* 22.4 (2007), pp. 1–20.

[6] D.S. Falconer. *Introduction to quantitative genetics.* 1960.
[7] J. Felsenstein. "Phylogenies and the Comparative Method". In: *American Naturalist* 125.1 (1985), pp. 1–15.
[8] C. Fraser et al. "Variation in HIV-1 set-point viral load: Epidemiological analysis and an evolutionary hypothesis". In: *Proceedings of the National Academy of Sciences of the United States of America* 104 (2007), pp. 17441–17446.
[9] R. P. Freckleton, P. H. Harvey, and M. Pagel. "Phylogenetic analysis and comparative data: A test and review of evidence". In: *American Naturalist* 160.6 (2002), pp. 712–726.
[10] L. Gras et al. "Viral load levels measured at set-point have risen over the last decade of the HIV epidemic in the Netherlands". In: *PLoS One* 4.10 (2009), e7365.
[11] J. T. Herbeck et al. "Is the virulence of HIV changing? A meta-analysis of trends in prognostic markers of HIV disease progression and transmission". In: *AIDS* 26.2 (2012), pp. 193–205.
[12] T. D. Hollingsworth, R. M. Anderson, and C. Fraser. "HIV-1 transmission, by stage of infection". In: *Journal of Infectious Diseases* 198.5 (2008), pp. 687–693.
[13] E. A. Housworth, E. P. Martins, and M. Lynch. "The phylogenetic mixed model". In: *American Naturalist* 163.1 (2004), pp. 84–96.
[14] T. Jombart and S. Dray. "adephylo: exploratory analyses for the phylogenetic comparative method." In: *Bioinformatics* 26 (2010), pp. 1907–1909. DOI: 10.1093/bioinformatics/btq292.
[15] S. W. Kembel et al. "Picante: R tools for integrating phylogenies and ecology". In: *Bioinformatics* 26.11 (2010), pp. 1463–1464.
[16] N. Mantel. "The detection of disease clustering and a generalized regression approach". In: *Cancer Res* 27.2 (1967), pp. 209–20.
[17] V. Müller et al. "Stable virulence levels in the HIV epidemic of Switzerland over two decades". In: *AIDS* 20.6 (2006), pp. 889–894.
[18] M. Pagel. "Inferring the historical patterns of biological evolution". In: *Nature* 401.6756 (1999), pp. 877–84.
[19] S. Pavoine et al. "Testing for phylogenetic signal in phenotypic traits: New matrices of phylogenetic proximities". In: *Theoretical Population Biology* 73.1 (2008), pp. 79–91.
[20] A. Rambaut. "Path-O-Gen: temporal signal investigation tool." In: (2010).
[21] L. J. Revell. *phylosig.* 2011.
[22] S. Y. Rhee et al. "Human immunodeficiency virus reverse transcriptase and protease sequence database". In: *Nucleic Acids Research* 31.1 (2003), pp. 298–303.
[23] R. W. Shafer. "Rationale and uses of a public HIV drug-resistance database". In: *Journal of Infectious Diseases* 194 Suppl 1 (2006), S51–8.
[24] R Development Core Team. *R: A language and environment for statistical computing.* 2011.
