# Supplementary material for: How effectively can HIV phylogenies be used to measure heritability?
Source: Evol Med Public Health. 2013 Sep 13;2013(1):209–24. doi: 10.1093/emph/eot019 (PMC3850537; doi:10.1093/emph/eot019)
Supplement: Supplementary Data [file supp_eot019_suppl_data.zip › Latex_bits.tar.gz!/Latex_bits/Master.pdf]

How effectively can HIV phylogenies be used to measure heritability?

George Shirreff[1,2], Samuel Alizon[3], Anne Cori[1], Huldrych F. Günthard[4], Oliver Laeyendecker[5,6], Ard van Sighem[7], Daniela Bezemer[7], and Christophe Fraser[1]

[1]Medical Research Council Centre for Outbreak Analysis and Modelling, Department of Infectious Disease Epidemiology, Imperial College, London, United Kingdom.
[2]Institute for Integrative Biology, ETH Zürich, Zürich, Switzerland
[3]Lab MIVEGEC UMR CNRS 5290, IRD 224, UM1, UM2, F-34394 Montpellier 5, France.
[4]Division of Infectious Diseases and Hospital Epidemiology, University Hospital of Zürich, University of Zürich, Zürich, Switzerland.
[5]National Institute of Allergy and Infectious Diseases, National Institutes of Health, Baltimore, Maryland, United States of America.
[6]Department of Medicine, Johns Hopkins University School of Medicine, Baltimore, Maryland, United States of America.
[7]Stichting HIV Monitoring, Amsterdam, Netherlands.

September 9, 2013

Abstract word count: 261
Article word count: 4799
Figures: 5
Tables: 3
Corresponding author: george.shirreff@env.ethz.ch, +41(0)446320659
Page heading: We explore the extent to which disease severity in HIV infections is under virus, rather than host, control. We develop statistical methods to detect and quantify heritability of virulence using phylogenetic relationships between viral sequences and apply these in diverse cohorts and to simulated data.

Contents

1 Abstract **3**

2 Background and Objectives **3**

3 Methodology **4**
3.1 Data 4
3.2 Methods for calculating heritability statistics 5
3.3 Randomisation test 6
3.4 Method of simulating SPVL data on a known phylogeny 6
3.5 Multiple Hypothesis Testing (MHT) to estimating true heritability and confidence intervals . 6
3.6 Estimate power to detect an effect 7

4 Results **7**
4.1 Testing significance in previously analysed data. 7
4.2 Estimates of heritability in previously analysed data 10

4.3 Testing the sensitivity of each statistic 12
4.4 Application to data from the Netherlands 12

5 Discussion **17**
5.1 Which cohorts exhibit heritability? 17
5.2 Which method is best for detecting heritability? 18
5.3 Conclusions and Implications 19

6 Acknowledgements **19**
6.1 Swiss HIV Cohort Study 19
6.2 Rakai Health Sciences Programme 20
6.3 ATHENA National Observational Cohort, Netherlands 20

Bibliography **24**

List of Figures

1 Heritability estimated by ABC in the all subdivisions of the Swiss data. 10
2 Heritability estimated by ABC in the all subdivisions of the Rakai data. 11
3 Comparison of the sensitivity of various statistics to heritability on the entire Swiss phylogeny. 13
4 Comparison of the sensitivity of phylogenetic comparative statistics to heritability on the Swiss MSM Strict phylogeny. 14
5 Heritability estimated by ABC in the all subdivisions of the Netherlands data. 16

List of Tables

1 Significance tests of heritability and confidence intervals of h^2 in the Rakai data. 8
2 Significance tests of heritability and confidence intervals of h^2 in the Swiss data. 9
3 Significance tests of heritability and confidence intervals of h^2 in the Netherlands data. 15

1 Abstract

Background and objectives
The severity of HIV-1 infection, measured by set-point viral load (SPVL), is highly variable between individuals. Its heritability between infections quantifies the control the pathogen genotype has over disease severity. Heritability estimates vary widely between studies, but differences in methods make comparison difficult. Phylogenetic comparative analysis offers measures of phylogenetic signal, but it is unclear how to interpret them in terms of the fraction of variance in SPVL controlled by the virus genotype.
Methodology
We present computational methods which link statistics summarizing phylogenetic signal to heritability, h^2 in order to test for and quantify it. We re-analyse data from Switzerland and Uganda, and apply it to new data from the Netherlands. We systematically compare established and new (e.g. Phylogenetic Pairs, PP) phylogenetic signal statistics.
Results
Heritability estimates varied by method and dataset. Several methods were consistently able to detect simulated heritability above $h^2 \approx 0.4$, but none below. Pagel's λ was the most robust and sensitive. The PP method found no heritability in the Netherlands data, while Pagel's λ found significant heritability only in a narrow subdivision (p=0.038). Heritability was estimated at $h^2 = 0.52$ (95% confidence interval 0.00-0.63).
Conclusions and implications
This standardised measure, h^2, allows comparability of heritability between cohorts. We confirm high heritability in Swiss data, but not in Ugandan data, nor in the Netherlands, where it is barely or undetectable. Existing phylogenetic methods are ill-suited for detecting heritability below $h^2 \approx 0.4$, which may nonetheless be biologically important.

2 Background and Objectives

HIV has a high mutation rate [31, 3] and daily turnover [27, 66] and therefore adapts rapidly under local selective pressure from the immune system [68, 8, 44] or antiretroviral drugs [51, 55]. Increasingly there is interest in the transmission of escape [37, 48] or drug resistance mutations [71], which may enable viral adaptation to the host population. The rate at which a trait evolves in response to natural selection is determined by its heritability.

Recent work has suggested that virulence may also evolve at the population level [26] by natural selection towards a level optimal for transmission [17, 56]. Variation in virulence has a large impact on mortality and morbidity, so its evolutionary potential may present challenges or provide opportunities in public health interventions [10]. For example, vaccines which reduce growth rate or toxicity are predicted to reduce the costs of virulence, to the vaccinated host and the pathogen. This potentially raises the optimal virulence, resulting in poorer outcomes for the unvaccinated individuals [20].

Virulence in HIV is well approximated by set-point viral load (SPVL), which refers to the density of virions in the blood during asymptomatic infection. SPVL is an early prognostic indicator for AIDS, as it varies by orders of magnitude between individuals, with high values having faster CD4 cell decline, progressing more rapidly to AIDS and death [67, 33, 40]. However, it is relatively stable within the individual [25] meaning it can be measured at a wide range of time points in an individuals infection [21].

Many host factors influence SPVL (HLA type (reviewed in [9]); sex [11]; ethnicity [42]; age [50]; co-infections [1, 32]). Specific human genetic markers have been identified to which 13% of SPVL variation can be attributed, with a further 9% explained by age, sex and population structure [14]. Recently, several studies have indicated that viral factors play a substantial role in SPVL variation by measuring its heritability between infections (reviewed in [41]). Most of these quantify the similarity in SPVL within transmission pairs, which are sexual couples in which one has infected the other [59, 24, 35, 29].

The phenotype of any organism is controlled partly by its genome, and partly by its environment. Throughout this work we define heritability, h^2, in the broad sense as the proportion of total phenotypic variance (${\sigma_P}^2$)

ascribed to genetic variance (${\sigma_G}^2$) (equation 1, [16]). In the environmental component of variance, ${\sigma_E}^2$, we conceptually include all host genetic and non-genetic effects, as well as interactions between host and virus genotype.

$$h^2 = \frac{\sigma_G^2}{\sigma_P^2} \tag{1}$$

Alizon et al. [2] used a phylogenetic comparative approach to identify phylogenetic signal as a measure of heritability, without requiring behavioural data. Phylogenetic signal is the extent to which individuals with similar traits can be observed to cluster together on the phylogeny. This approach has the advantage that any sample of well-characterised patients could be analysed in this way. However, the authors did not account for cofactors such as age and co-infections which influence the SPVL and may cluster together on the phylogeny. It is also uncertain exactly how the quantity measured by the two methods used (Pagel's λ and Blomberg's K) should be interpreted in the context of heritability. We herein propose a new approach which also uses only the phylogenetic relationships to determine genetic proximity of viruses, but additionally links the results to true heritability, and in some cases allows inclusion of the effect of cofactors on SPVL.

The aims of this study were: to evaluate the phylogenetic approach for estimating heritability; to compare the efficacy of the various statistics available for quantifying heritability on simulated and real data; to use these methods to confirm the presence of heritability in previously analysed data; and to measure heritability in a dataset from the Netherlands which has not been previously analysed for this purpose.

3 Methodology

3.1 Data

Data from Rakai, Uganda

The study population was enrolled in the Rakai Community Cohort Study in the rural Rakai District of south-western Uganda. The study methods for this cohort have been outlined elsewhere [64, 65, 29]. We used those individuals who were sampled at a single time point in April 1995 ($n = 332$). Characteristics of the cohort are shown in Table S1. Serum samples were used to measure SPVL, with most individuals providing a single measurement.

The phylogenetic analysis was conducted in RAxML [57] using the General Time Reversible substitution model [60], the Γ model of rate heterogeneity with 4 distinct rate categories, and a proportion of invariant sites [70] (GTR+Γ+I), which had the best score when the alignment was analysed with ModelTest [49]. The alignment was also analysed using the Recombination Analysis Tool (RAT [12]), which found no apparent recombination within the studied genes (data not shown).

An SIV outgroup was included (Accession number: AB177846.1). The phylogeny was constructed from either the *gp41* (*env*) and *p24* (*gag*) sequences (Figures S1 and S2) which were analysed separately because they have different substitution rates [43]. We also performed the phylogenetic and heritability analysis on the subtype A and D sequences separately, due to apparent imbalance in the joint trees.

Data from Switzerland

The Swiss data was taken from the Swiss HIV Cohort Study and its integrated genotypic drug resistance database, which has been described elsewhere [36, 54] and the specific selection has been analysed in previous work [2, 34, 69]. The phylogeny, constructed from the reverse transcriptase and protease components of the *pol* gene (Figure S3), had been inferred in this previous work [2] using PhyML [23] from subtype B infected individuals for whom at least three viral load measurements were available after primary infection and before anti-retroviral therapy ($n = 661$). Characteristics of the cohort are shown in Table S2.

Note that PhyML was used for the Swiss data, compared with RAxML in the other two datasets. However, we expect the methods to produce very similar results as both use a hill-climbing algorithm, likelihood scores are well matched when using the same nucleotide substitution model, and there is no evidence for systematic differences in the results [58].

We analysed four subdivisions of the data as in previous work [2]. The entire dataset ("All") fit the Liberal definition for viral load variability, meaning that at least three consecutive viral load measurements within the asymptomatic window (6-36 months after first positive viral RNA) remained within a one-log band of one another. The "Strict" subdivision included only those individuals for whom the measurements in the asymptomatic window all sat within the one-log band. Excluding all but men who have sex with men (MSM) led to the further subdivisions, "MSM" and "MSM Strict".

Data from Netherlands

The study cohort was provided by the ATHENA national observational cohort (seroconverters from 1996 or onwards) and the Amsterdam Cohort Studies (seroconverters before 1996) [22]. We included only individuals infected with subtype B between 1985 and 2008, for whom appropriate genetic data and SPVL were available [6] ($n = 416$).

We define SPVL as the mean of all $\log_{10}$ viral load measurements taken 6-24 months after the midpoint between the last negative and first positive diagnosis. The phylogeny was reconstructed using a sequence of length 2064 containing the same elements of the *pol* gene as for the Swiss data.

We excluded codons strongly associated with drug resistance mutations (for details see Figure S4). Four subtype C individuals from the same cohort were used as the outgroup. The phylogenetic analysis was performed using ModelTest [49], RAxML [57] and RAT [12] in the same way as the Rakai cohort, which also identified the same nucleotide substitution model as appropriate, and no recombination was detected (data not shown).

As with the Swiss data, individuals were categorised as "All", "Strict", "MSM" and "MSM Strict". Additionally, two further categories, "MSM NL" and "MSM Strict NL", were created from the "MSM" groups, which excluded individuals not originating in the Netherlands in order to further reduce confounding factors.

Trees for simulations were read and manipulated using the *ape* package [45] in R [61], which was also used to plot the trees.

3.2 Methods for calculating heritability statistics

A phylogeny, reconstructed from genetic data, is an approximation of the transmission network. Phylogenetic signal is a measure of how well trait values at the tree tips match their relative positions on the phylogeny, and several established methods are available to quantify this signal in terms of a single statistic: the Mantel test [38]; Blomberg's independent contrasts, which give us the Blomberg's K and PICv statistics [7]; Pagel's λ transformation [19]; and the Abouheif-Moran (AM) tests[47], of which there are five variants ('oriAbouheif', 'sumDD', 'nNodes', 'patristic', 'Abouheif', with the latter used by default). We also developed two new methods which allow control of cofactors, the Phylogenetic Pairs method, and the Hierarchical Clustering method, which are described briefly here.

The Phylogenetic Pairs (PP) method identifies pairs of individuals on the tree which are each other's closest neighbour, and these are assumed to be transmission pairs. Analysis of variance (ANOVA) identifies the degree to which the transmission partner explains an individual's SPVL. Crucially, the ANOVA approach allows for the inclusion of cofactors. These are age, sex and genital ulcer disease (GUD) in the Rakai dataset (Table S1); age, sex and risk group in the Swiss dataset (Table S2); and age, sex, risk group, region of origin and the type of assay used to measure viral load in the Netherlands dataset (Table S3). This method also ignores individuals who are not part of a phylogenetic pair.

The Hierarchical Clustering (HC) method is similar but considers larger clusters of individuals identified on the phylogeny by a threshold branch length, and examines the amount of variance in SPVL explained by the cluster. Because there is no intuitive ideal cluster size, proportion included or number of clusters to use, the method integrates over the range of clustering distances.

All established and new methods are described in detail in the Supplementary Information (6.3).

3.3 Randomisation test

The significance of a test statistic can be measured as in [7] by comparing the statistic derived from the data with its distribution under no heritability, which is the null hypothesis. Randomising the tips of the tree by randomly reallocating the tips scrambles any heritability signal. This is performed 1000 times, and the analysis is repeated for each. The proportion of randomised datasets which give a statistic higher than the true value is the one-tailed p-value for presence of heritability. When the randomisation test was performed for the PP and HC statistics the cofactors remained with their corresponding SPVL data.

3.4 Method of simulating SPVL data on a known phylogeny

This method uses a simple algorithm to simulate evolution of a continuous trait on a known phylogeny for a given heritability. This method is similar to that used in previous work [2] and is a variation on the Ornstein-Uhlenbeck process [62, 15] which allows Brownian motion to occur while constraining the distribution of the population.

During the simulation, each node on the tree is assigned a trait value in $\log_{10}$ SPVL, beginning from the root, which is assigned the mean of the true SPVL data. Each daughter node is given a SPVL value depending on that of its parent, and on the SPVL distribution in the whole population. The higher the heritability, the more the value depends on the parent.

The SPVL at each subsequent daughter node, V_D (equation 2), is derived from the parent node, V_P, the value h^2, and the random variable M which is normally distributed according to the mean and variance of the population $\log_{10}$ SPVL (distribution 3).

$$V_D = h^2 V_P + M\sqrt{1-(h^2)^2} \tag{2}$$

$$M \sim N(\bar{\mu}, {\sigma_P}^2) \tag{3}$$

The value h^2 is therefore the regression slope between the trait values at a parent node, V_P, and a daughter node, V_D (or an index and secondary case). This has been demonstrated to be equal to the broad sense heritability, h^2 as defined in equation 1 [13], and its further implications are discussed in the Supplementary Information (6.3). The result is a set of data at the tips of the tree, simulated with known heritability h^2, which is used for analysis. We have also explored an alternative method of simulation which allows for multiple transmission between nodes on the tree, which is also described in the Supplementary Information (6.3).

3.5 Multiple Hypothesis Testing (MHT) to estimating true heritability and confidence intervals

For each value of h^2 between 0 and 1, in increments of 0.01, 100 simulations are performed and the relevant phylogenetic comparative statistics calculated. A hypothesis test for the particular h^2 is then performed with these 100 values. They are compared with the statistic calculated from the true data, and the proportion which are lower than the true statistic becomes the probability p that the data are consistent with that value

of h^2. The values of h^2 which produce p values closest to 0.025, 0.975 and 0.500 become the lower and upper 95% confidence bounds, and the median estimate of h^2, respectively.

A visual distribution of h^2 is estimated using Approximate Bayesian Computation (ABC), of which details are given in the Supplementary Information (6.3).

3.6 Estimate power to detect an effect

To examine the ability of each statistic to detect non-null heritability, we simulated 100 datasets for each value of a range (0-1) of h^2 values in increments of 0.05. To visualise the relationship between h^2 and the statistic of interest, we calculated the mean and standard deviation of the phylogenetic comparative statistics calculated from these 100 simulations. In order to estimate the power of each statistic to detect a significant effect at each value of h^2, we performed a randomisation test on each simulated dataset, as described above (3.3), but with 100 randomisations. For each simulated dataset, the proportion of randomisation tests which find significant ($p < 0.05$) heritability represents the power to detect an effect at that value of h^2.

All methods are available in R code on request from the corresponding author.

4 Results

4.1 Testing significance in previously analysed data.

The significance of heritability from the randomisation test for the Rakai and Swiss data are shown in Tables 1 and 2 respectively.

A consistent signal was not found in the Rakai data (Table 1), but in the Swiss data it was more so (Table 2), and was higher for the smaller subdivisions. We tested the possibility that high significance in the smallest group was an artifact of small tree size, but this was not the case (Supplementary Information 6.3). We also tested the robustness of the results to phylogenetic uncertainty and found that small levels of perturbation (3%) have little effect on the results, but as the level of perturbation increases, the signal weakens accordingly.

Table 1: The statistics (Z), p-values from a randomisation test, and confidence intervals of h^2 from multiple hypothesis testing on the Rakai data. This included from analysis of the *gp41* and *p24* genes in all the available Rakai data, or subtypes A or D separately. P-values showing borderline significance (p<0.1) are in blue, and formal significance (p<0.05) is in red, and confidence intervals in which the lower limit is above zero are also in red.

	gp41 (n=325)				subtype A *gp41* (n=46)				subtype D *gp41* (n=214)				*p24* (n=327)				subtype A *p24* (n=47)				subtype D *p24* (n=217)			
	Z	p	h^2	95% CI	Z	p	h^2	95% CI	Z	p	h^2	95% CI	Z	p	h^2	95% CI	Z	p	h^2	95% CI	Z	p	h^2	95% CI
PP	0.490	0.050	0.75	(0.00, 0.84)	−0.095	0.619	0.17	(0.00, 0.74)	0.311	0.340	0.66	(0.00, 0.83)	−0.099	0.668	0.08	(0.00, 0.56)	−0.089	0.633	0.02	(0.00, 0.81)	0.358	0.125	0.64	(0.00, 0.83)
HC complete	0.024	0.149	n.d.	n.d.	−0.001	0.514	n.d.	n.d.	0.029	0.056	n.d.	n.d.	−0.006	0.695	n.d.	n.d.	0.002	0.464	n.d.	n.d.	0.010	0.188	n.d.	n.d.
HC single	0.003	0.350	n.d.	n.d.	0.003	0.261	n.d.	n.d.	0.001	0.411	n.d.	n.d.	−0.003	0.670	n.d.	n.d.	−0.005	0.736	n.d.	n.d.	0.000	0.407	n.d.	n.d.
HC average	0.016	0.181	n.d.	n.d.	0.002	0.428	n.d.	n.d.	0.022	0.056	n.d.	n.d.	−0.008	0.783	n.d.	n.d.	0.001	0.477	n.d.	n.d.	0.017	0.040	n.d.	n.d.
HC ward	0.037	0.111	0.49	(0.00, 0.67)	−0.011	0.653	0.07	(0.00, 0.68)	0.041	0.038	0.59	(0.00, 0.78)	−0.011	0.756	0.08	(0.00, 0.49)	−0.004	0.555	0.08	(0.00, 0.73)	0.011	0.244	0.44	(0.00, 0.66)
Mantel	−0.018	0.779	0.72	(0.00, 0.95)	0.174	0.034	0.56	(0.00, 0.80)	−0.007	0.547	0.02	(0.00, 0.95)	−0.006	0.569	0.57	(0.00, 0.99)	0.024	0.392	0.39	(0.00, 0.59)	0.016	0.362	0.92	(0.00, 0.96)
Blomberg K	0.001	0.705	0.09	(0.00, 0.59)	0.861	0.044	0.47	(0.00, 0.60)	0.001	0.737	0.06	(0.00, 0.54)	0.724	0.050	0.47	(0.00, 0.60)	0.452	0.160	0.43	(0.00, 0.58)	0.563	0.250	0.38	(0.00, 0.56)
PICv	3101	0.335	n.d.	n.d.	11.5	0.797	n.d.	n.d.	4101	0.340	n.d.	n.d.	15.6	0.858	n.d.	n.d.	27.6	0.578	n.d.	n.d.	16.2	0.758	n.d.	n.d.
Pagel λ	0.473	0.275	0.25	(0.00, 0.58)	0.624	0.051	0.50	(0.00, 0.61)	1.55	0.169	0.87	(0.00, 1.00)	0.700	0.011	0.51	(0.02, 0.61)	0.000	0.746	0.10	(0.00, 0.54)	0.000	0.424	0.31	(0.00, 0.55)
AM oriAbouheif	−0.012	0.640	n.d.	n.d.	−0.030	0.614	n.d.	n.d.	0.022	0.301	n.d.	n.d.	0.045	0.107	n.d.	n.d.	0.014	0.426	n.d.	n.d.	0.024	0.295	n.d.	n.d.
AM Abouheif	−0.013	0.613	0.04	(0.00, 0.41)	−0.113	0.842	0.15	(0.00, 0.48)	0.019	0.277	0.20	(0.00, 0.49)	0.035	0.152	0.26	(0.00, 0.51)	−0.041	0.562	0.08	(0.00, 0.56)	0.021	0.291	0.29	(0.00, 0.51)
AM sumDD	−0.003	0.525	n.d.	n.d.	−0.046	0.799	n.d.	n.d.	−0.001	0.212	n.d.	n.d.	0.001	0.186	n.d.	n.d.	−0.031	0.629	n.d.	n.d.	−0.002	0.328	n.d.	n.d.
AM nNodes	−0.003	0.525	n.d.	n.d.	−0.046	0.799	n.d.	n.d.	−0.001	0.212	n.d.	n.d.	0.001	0.186	n.d.	n.d.	−0.031	0.629	n.d.	n.d.	−0.002	0.328	n.d.	n.d.
AM patristic	0.015	0.027	n.d.	n.d.	−0.021	0.356	n.d.	n.d.	0.020	0.030	n.d.	n.d.	−0.003	0.498	n.d.	n.d.	−0.018	0.368	n.d.	n.d.	−0.005	0.486	n.d.	n.d.

PP – Phylogenetic Pairs
HC – Hierarchical Clustering
PICv – Variance of Phylogenetic Independent Contrasts
AM – Abouheif Moran tests
n.d. – not done

Table 2: The statistics (Z), p-values from a randomisation test, and confidence intervals of h^2 from multiple hypothesis testing on the Swiss data. Individuals were subdivided according to variability of viral load (Strict), risk category (MSM) or both.

P-values showing borderline significance (p<0.1) are in blue, and formal significance (p<0.05) is in red, and confidence intervals in which the lower limit is above zero are also in red.

	All (n=661)				Strict (n=230)				MSM (n=404)				MSM Strict (n=134)			
	Z	p	h^2	95% CI	Z	p	h^2	95% CI	Z	p	h^2	95% CI	Z	p	h^2	95% CI
PP	−0.089	0.817	0.08	(0.00, 0.48)	0.376	0.020	0.61	(0.01, 0.77)	0.009	0.492	0.18	(0.00, 0.53)	0.595	0.005	0.68	(0.12, 0.82)
HC complete	0.008	0.214	n.d.	n.d.	0.045	0.038	n.d.	n.d.	0.020	0.096	n.d.	n.d.	0.049	0.032	n.d.	n.d.
HC single	0.006	0.159	n.d.	n.d.	0.030	0.038	n.d.	n.d.	0.008	0.175	n.d.	n.d.	0.034	0.040	n.d.	n.d.
HC average	0.004	0.315	n.d.	n.d.	0.039	0.043	n.d.	n.d.	0.016	0.113	n.d.	n.d.	0.046	0.024	n.d.	n.d.
HC ward	0.008	0.315	0.36	(0.00, 0.52)	0.060	0.020	0.58	(0.28, 0.72)	0.036	0.036	0.44	(0.00, 0.61)	0.063	0.023	0.62	(0.34, 0.73)
Mantel	−0.014	0.712	0.00	(0.00, 0.80)	0.040	0.201	0.91	(0.00, 0.97)	−0.005	0.516	0.00	(0.00, 0.76)	0.050	0.207	0.79	(0.00, 0.91)
Blomberg K	0.002	0.305	0.50	(0.00, 0.67)	0.025	0.366	0.44	(0.00, 0.72)	0.091	0.038	0.71	(0.00, 0.82)	0.593	0.080	0.42	(0.00, 0.54)
PICv	1594	0.307	n.d.	n.d.	127	0.345	n.d.	n.d.	40.2	0.052	n.d.	n.d.	5.58	0.002	n.d.	n.d.
Pagel λ	0.105	0.016	0.44	(0.16, 0.57)	0.216	0.030	0.44	(0.00, 0.57)	0.153	0.025	0.45	(0.00, 0.59)	0.646	0.016	0.54	(0.32, 0.63)
AM oriAbouheif	−0.013	0.680	n.d.	n.d.	0.016	0.361	n.d.	n.d.	0.038	0.141	n.d.	n.d.	0.127	0.023	n.d.	n.d.
AM Abouheif	−0.014	0.671	0.01	(0.00, 0.32)	0.015	0.338	0.24	(0.00, 0.49)	0.037	0.138	0.31	(0.00, 0.48)	0.126	0.019	0.49	(0.03, 0.61)
AM sumDD	0.000	0.166	n.d.	n.d.	0.001	0.168	n.d.	n.d.	0.001	0.136	n.d.	n.d.	0.013	0.016	n.d.	n.d.
AM nNodes	0.000	0.166	n.d.	n.d.	0.001	0.168	n.d.	n.d.	0.001	0.136	n.d.	n.d.	0.013	0.016	n.d.	n.d.
AM patristic	0.002	0.320	n.d.	n.d.	0.002	0.155	n.d.	n.d.	0.005	0.119	n.d.	n.d.	0.000	0.054	n.d.	n.d.

PP – Phylogenetic Pairs
HC – Hierarchical Clustering
PICv – Variance of Phylogenetic Independent Contrasts
AM – Abouheif Moran tests
n.d. – not done

4.2 Estimates of heritability in previously analysed data

Tables 1 and 2 show the medians and confidence intervals of h^2 as estimated by MHT. In the Swiss data the confidence intervals exclude zero in several cases. In all of these instances the randomisation test was also significant. However, a significant randomisation test did not always correspond to confidence intervals which excluded zero, because these are one- and two-tailed tests, respectively.

The distribution of true heritability was calculated by ABC using both Phylogenetic Pairs (red) and Pagel's λ (blue) for each subdivision in the Swiss and Rakai datasets (Figures 1 and 2). In the Swiss figure, the MSM Strict subdivision exhibits the distribution of h^2 most removed from zero, which supports the results from the randomisation test and lower confidence bounds (Table 2). The method using Pagel's λ exhibits higher heritability in the larger subdivisions.

Similarly in the Rakai cohort, the most significant results from the randomisation test (Table 1) is the *p24* subdivision analysed under Pagel's λ, and this is reflected in the results from ABC, in which the distribution of estimated h^2 is highly positive. Interestingly, the Phylogenetic Pairs method finds that the most significant distribution visually is instead from the *gp41* subdivision which is also confirmed by the randomisation test.

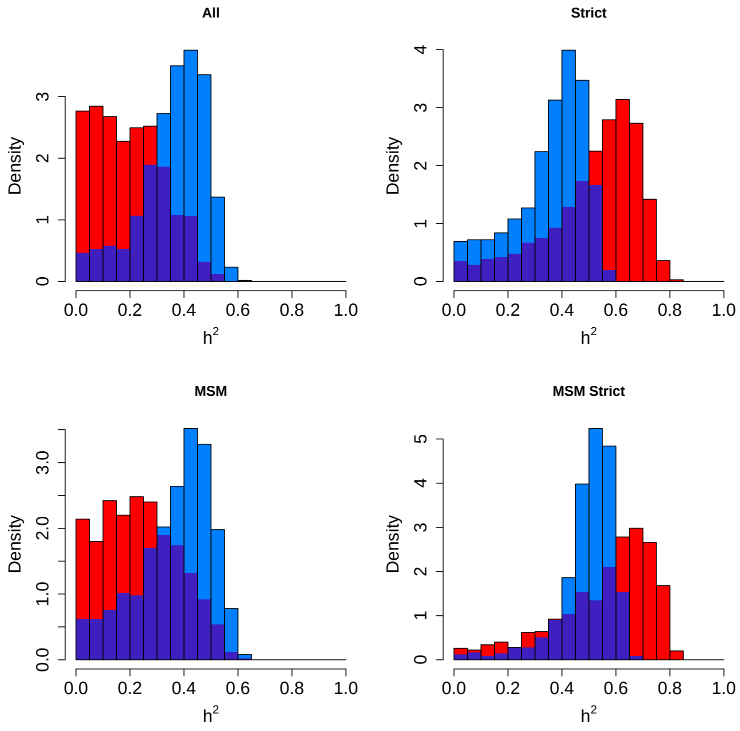

Figure 1: Heritability estimated by ABC in all subdivisions of the Swiss data. The subdivision is written above the plot. Results from the Phylogenetic Pairs method are in red, and from Pagel's λ in blue, with the overlap in purple.

We also tested an alternative method of simulation which accounted for differences in branch length and allowed multiple generations between two adjacent nodes. We applied this to the Rakai *p24* subtype A data (Supplementary Table S7), and the alternative gave higher estimates of h^2 but wider confidence intervals.

Heritability was also measured in the Rakai and Swiss data using a Phylogenetic Mixed Model, which assumes that the trait is determined by independent viral and host effects [30]. These results are given in the Supplementary Information, Table S4.

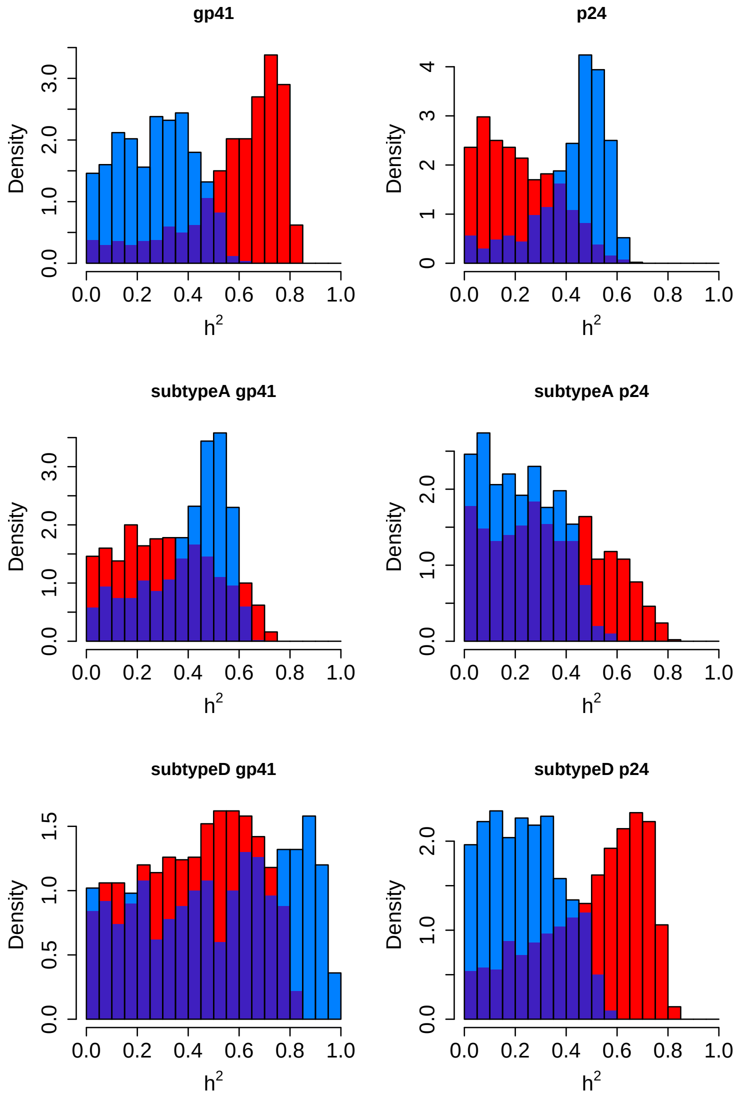

Figure 2: Heritability estimated by ABC in all subdivisions of the Rakai data. The subdivision is written above the plot. Results from the Phylogenetic Pairs method are in red, and from Pagel's λ in blue, with the overlap in purple.

4.3 Testing the sensitivity of each statistic

The sensitivity of each phylogenetic comparative statistic to simulated heritability was explored by visualizing the relationship between them, and measuring the power of the statistic to successfully detect an effect of that size, in the entire Swiss dataset and the MSM Strict subdivision (Figures 3 to 4, respectively). None of the statistics had substantial power to detect heritability below h^2=0.4 on any subdivision, but most had the power to detect an effect above that level. The Mantel test performed poorly throughout. Blomberg's K performed well in the MSM Strict subdivision, but was insensitive when applied to the entire Swiss dataset, with only values above h^2=0.6 being consistently detected. In the entire Swiss dataset, the AM statistic appeared the most sensitive, but in the MSM Strict subdivision its performance matched that of Pagel's λ.

We also tested the power of variants of the HC and AM tests compared with their default methods ('ward' and 'Abouheif' respectively) (Supplementary Figures S5 and S6). The default methods performed equally well or better than any of the variations.

4.4 Application to data from the Netherlands

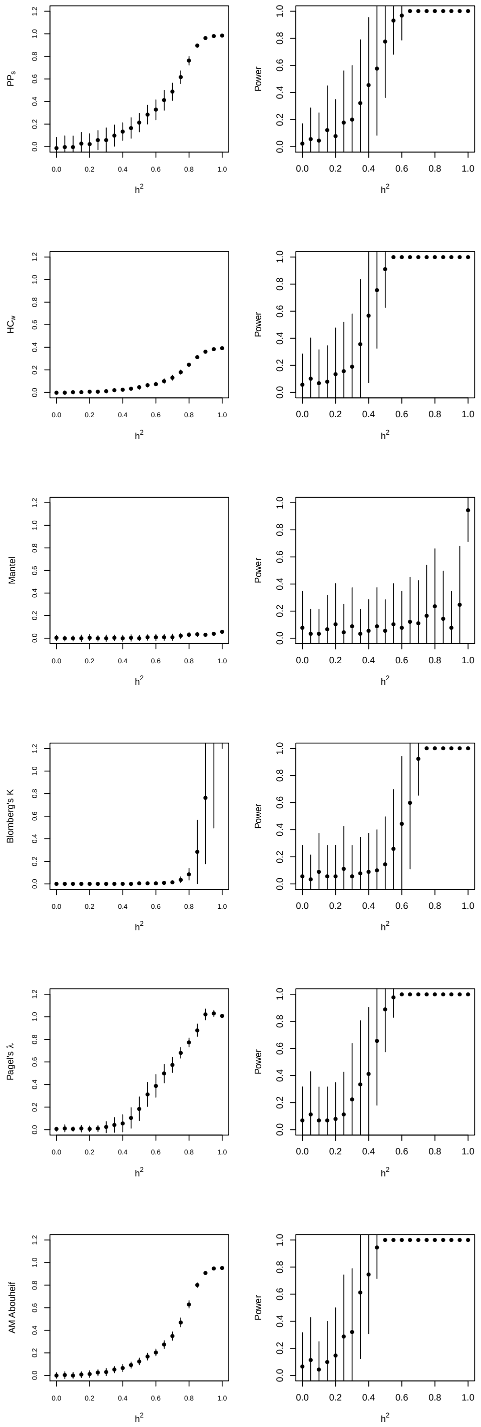

Figure 3: Comparison of the sensitivity of various statistics to heritability on the entire Swiss phylogeny. (Left side) The relationship between heritability and six different statistics under comparison. The circles and bars represent the mean and standard deviation of the sample. (Right side) The power of each statistic to detect heritability at 5% significance. The bars represent the standard deviation of the proportion.

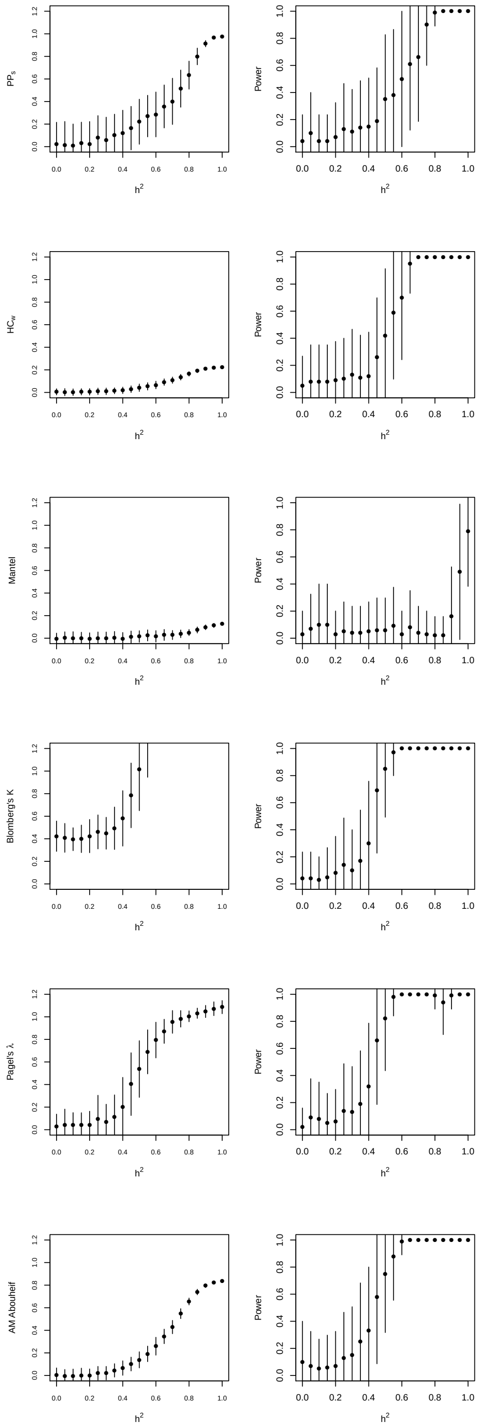

Figure 4: Comparison of the sensitivity of phylogenetic comparative statistics to heritability on the Swiss MSM Strict phylogeny. (Left side) The relationship between heritability and six different statistics under comparison. The circles and bars represent the mean and standard deviation of the sample. (Right side) The power of each statistic to detect heritability at 5% significance. The bars represent the standard deviation of the proportion.

Table 3: The statistics (Z), p-values from a randomisation test, and confidence intervals of h^2 from multiple hypothesis testing on the Netherlands data. Individuals were subdivided according to variability of viral load (Strict), risk category (MSM) or whether they were from the Netherlands (NL). P-values showing borderline significance ($p<0.1$) are in blue, and formal significance ($p<0.05$) is in red, and confidence intervals in which the lower limit is above zero are also in red.

		All	Strict	MSM	MSM Strict	MSM NL	MSM NL Strict
	n	416	246	348	211	286	174
PP	Z	-0.0627	0.2193	0.0763	0.2047	0.0633	0.2277
	p-value	0.703	0.111	0.272	0.128	0.327	0.130
	h^2	0.02	0.01	0.08	0.09	0.29	0.40
	95% CI	(0.00,0.45)	(0.00,0.56)	(0.00,0.53)	(0.00,0.47)	(0.00,0.63)	(0.00,0.67)
Pagel's λ	Z	0.0001	0.0978	0.0001	0.0323	0.0001	0.2628
	p-value	0.824	0.086	0.827	0.200	0.718	0.038
	h^2	0.19	0.44	0.01	0.38	0.10	0.52
	95% CI	(0.00,0.50)	(0.00,0.60)	(0.00,0.51)	(0.00,0.60)	(0.00,0.55)	(0.00,0.63)

PP – Phylogenetic Pairs

Supplementary Figure S4 shows the shape of the phylogeny inferred from the Netherlands data. These data have not been previously analysed for heritability, and so to reduce the problem of multiple testing only two methods, Pagel's λ and Phylogenetic Pairs (PP), were used to detect and measure heritability in each subdivision of the data.

Significant heritability was found only in one subdivision (MSM from the Netherlands with Strict viral loads) and using one statistic (Pagel's λ), which gave a heritability estimate of h^2=0.52 (Table 3). No effect was found when the PP method was used. None of the confidence intervals on h^2 excluded 0. The estimated distributions of h^2 are shown in Figure 5.

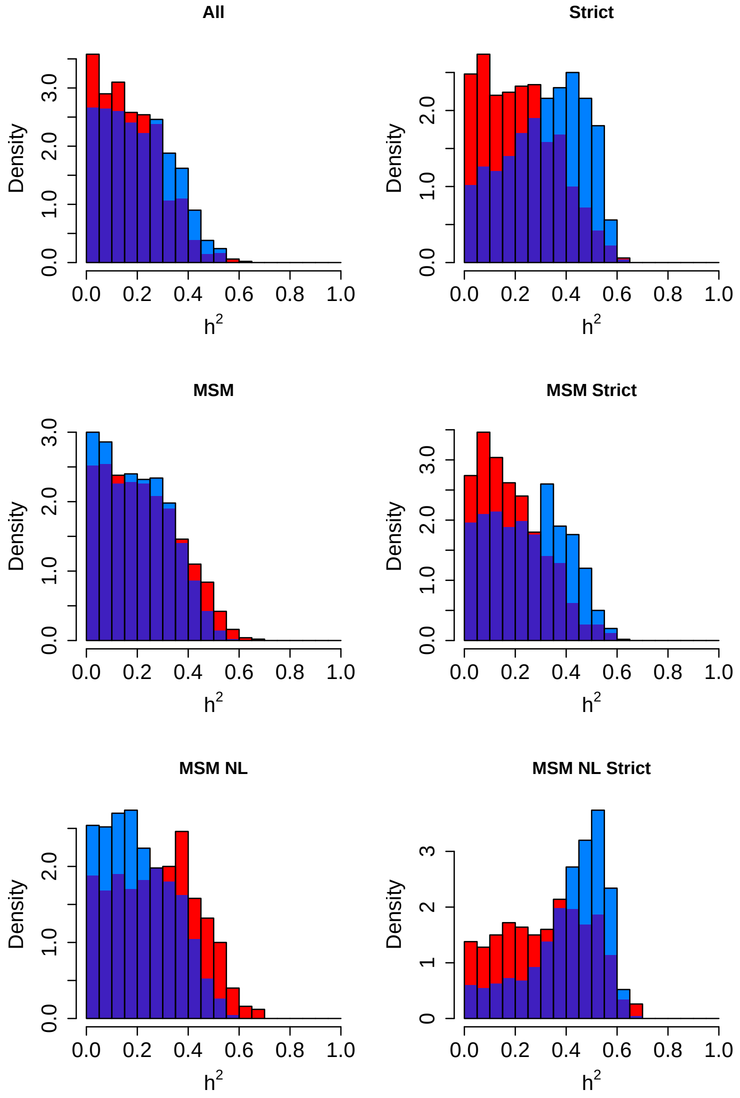

Figure 5: Heritability estimated by ABC in all subdivisions of the Netherlands data. The subdivision is written above the plot. Results from the Phylogenetic Pairs method are in red, and from Pagel's λ in blue, with the overlap in purple.

5 Discussion

In this study we developed new methods for detecting and measuring heritability of SPVL using phylogenetic relationships and compared them with established methods on real and simulated data.

5.1 Which cohorts exhibit heritability?

Heritability was detected consistently in the MSM Strict subdivision of the Swiss cohort (Table 2), supporting the previous study of this data which found significant and high heritability in that subdivision [2]. Note that in that study, the level of significance was detected by randomizing the PICv rather than the K statistic, hence the strong result for PICv in the MSM Strict subdivision. We also found that several statistics uncovered significant heritability in all of the subdivisions of the Swiss data. In many such cases, the confidence intervals of h^2 also excluded zero, and the estimates for h^2 were high (0.44 to 0.68).

In spite of significant heritability being found in previous work on the Rakai data [29], we did not consistently find heritability in this cohort. Although they are not strictly comparable, the results from the previous studies of the Rakai and Swiss data suggest that heritability is higher in the latter cohort, and this may underly the differences in our results. However, the best estimate for h^2 from the Rakai data, was high (0.51) (Table 1).

Six different subdivisions of the Netherlands data were analysed using PP and Pagel's λ, and only a single positive result was found with one statistic (λ) in one subdivisions, suggesting that heritability is close to the detection borderline. It was estimated at h^2=0.52 (Table 3), which falls high within the range of previous estimates [41].

It is interesting that the Swiss cohort shows more apparent heritability than the Netherlands in spite of a similar transmission routes and genetic backgrounds. One potential explanation would be differences in coverage. The prevalence in Switzerland is higher (0.4% compared with 0.2%) [63], but so is the sample size (661 compared with 416) which suggests similar levels of coverage, which are reportedly high in both cohorts [34, 5]. In the Swiss dataset, the mean SPVL of the Strict group is significantly higher than that of the rest (p=0.0004), and the same is true of the MSM over non-MSM (p=0.003) [2]. The same is not true of these two categories in the Netherlands cohort (data not shown), which suggests that viral virulence genotypes are less structured in this cohort and may explain lower heritability.

The exclusion of non-Strict individuals generally increased the level of significance, but this was dependent on the cohort and the method. In the Swiss cohort, the exclusion increased significance when using the PP and HC methods, but not otherwise (Table 2). In the Netherlands cohort, the exclusion increased significance markedly when using Pagel's λ, but not as much using PP (Table 3). Understanding why the Strict population exhibits higher heritability than whole sample may be an important step in both estimating and understanding the mechanisms behind heritability. Simple models fitted to longitudinal viral load data within patients suggest that fluctuations do not just represent random noise [39]. Fluctuating viral load has been associated with untreated sexually transmitted co-infections [4] and this or other host-mediated effect may mask the effect of the viral genotype and justify the exclusion of individuals with highly variable viral loads.

The same gene *pol* was used to analyse the Swiss and Netherlands datasets, whereas the available genes from the Rakai cohort were *env* and *gag*. It has been shown that *env* and *pol* produce similar trees [52], but there are topological differences, and our work also demonstrates different results between *gag* and *env* (Figure 2). The ABC method of estimation by simulation makes estimation of h^2 robust to differences in gene usage and we do not expect systematic differences between the cohorts. There are also differences in transmission routes between the cohorts, and a higher diversity of HLA types in African than European populations [53]. Although these differences inhibit direct comparability between the European and Ugandan datasets, they add to the general nature of this work.

5.2 Which method is best for detecting heritability?

The principle test of these statistics is the detection of an effect in real data. Pagel's λ detected heritability in every subdivision of the Swiss data (Table 2), and also produced the strongest result of any statistic applied to the Rakai dataset (Table 1). The PP and HC statistics performed well on the Swiss data, particularly on the Strict subdivisions. The AM statistics were less successful at detecting an effect in the Swiss data. In the Rakai data, the AM 'patristic' variant found a significant result in two subdivisions. However, in simulation studies it performed very poorly (Supplementary Figure S6).

Testing the detection power by simulation relies on a simple model of trait evolution, but has the advantage that heritability is known. It revealed that the PP, HC, Pagel's λ and AM statistics were comparably sensitive, detecting an effect at greater than approximately 0.4 heritability in the Swiss data, with AM slightly more sensitive (Figures 3 and 4). The K statistic was as sensitive as other statistics when applied to the MSM Strict subdivision, but in the entire Swiss dataset its sensitivity was low.

Simulations suggest that none of the methods can detect heritability lower than approximately h^2=0.4, and this threshold is higher in some phylogenies. This threshold is confirmed by the finding that estimates of h^2 are always above 0.4 when heritability was found to be significant. Most studies have estimated lower heritability than this [41], and previous modelling work has suggested that such low heritability is enough to produce a substantial rate of evolution [56]. This suggests that phylogenetic methods are not adequate to exclude the possibility of relevant heritability in HIV virulence in these datasets.

Interestingly, another study which took an analytical and computational approach to comparing between Blomberg's K, the AM and the Mantel test found that K had a higher power to detect an effect than the AM statistic [46]. They also argue that these tests should all give the same significance as they are based on the cross-product of a phylogenetic similarity and trait similarity matrix. In contrast, we found marked differences between their performances, with the AM, K and Mantel statistic having decreasing power to detect an effect. They found that the sensitivity of these methods was dependent on the shape of the phylogeny, so differences in the source of trees (simulated vs inferred) is a possible source of discrepancy in the respective studies. This is beyond the scope of the current paper but deserves to be the subject of future work.

The PP, along with the HC methods, is able to account for cofactors of SPVL: ignoring these may lead to overestimates or underestimates of heritability if they associate or dissociate on the tree, respectively. Indeed, an increase in signal when cofactors were taken into account was seen in previous analysis of transmission pairs in the Rakai cohort [29]. However, in the absence of a method to include the effect of cofactors in simulations, this aspect of the PP or HC methods cannot be harnessed to measure h^2. One possible method would be to calculate the fixed effects of the cofactors using a linear regression, simulate only the residuals, and subsequently add the fixed effects, but this requires treating residuals as data, which is inappropriate in the likely event that there is correlation between the effects of cofactors and the SPVL [18]. The PP method produces a dataset of couples, which is analogous to other couples studies [59, 29, 24, 35]. The lack of sensitivity of the PP method and the other phylogenetic methods suggests that the phylogeny cannot (yet) tell us everything that the epidemiology does about the transmission network.

The λ statistic has the advantage that it incorporates both topology and branch lengths, and analyses the entire sample. It is notable, therefore, that the PP method is relatively successful in spite of its analyzing only a subset of individuals who form apparent transmission pairs (60%), and in particular ignores deep relationships within the phylogeny. This suggests that most signal lies in the recent phylogenetic relationships. However, in unpublished work, Hodcroft et al. found SPVL heritability using pedigree analysis on UK data [28]. In contrast to our work, they found that collapsing poorly supported nodes in the tree and thereby ignoring some of the shallow relationships in the phylogeny, had a negligible effect on their results for some datasets. The AM method is also successful, which (with the exception of the 'patristic' variant) ignores branch lengths, indicating that topology may be more important. Rigorously identifying which clades or levels of the phylogeny are responsible for heritability would be an interesting direction for future research. This may differ for phylogenies which are less star-like than HIV. The use of longer sequences and better sampled datasets is likely to result in better detection and estimation of heritability, as poorly resolved trees

scramble the heritability signal. However, the detection threshold is unlikely to change even with improved sampling, as it was based on simulations which were blind to uncertainty in the tree.

It is noteworthy that the PP and Pagel's λ each have their strengths in estimating the distributions of h^2 in the different Rakai subdivisions. The PP method identifies a more strongly positive distribution in subdivision *gp41*, which Pagel's λ does not, but the latter detects a more positive distribution in *p24* and subtype A *gp41*. This suggests that an approach which combines these methods may be appropriate.

5.3 Conclusions and Implications

In this study we compare several phylogenetic comparative methods to detect heritability, h^2. Many methods detect heritability successfully in real and simulated data, but sensitivity drops off below h^2=0.4. We recommend the Phylogenetic Pair method and Pagel's λ for use in detecting and estimating heritability, the former for its consideration of co-factors, and the latter for its marginally higher level of sensitivity.

Estimates of heritability were consistent with previous studies on the Rakai and Swiss data, and confirm that heritability can be very high, which has clinical and evolutionary implications. When applied to Netherlands data, heritability was found only in the most homogeneous subdivision, MSM who originate in the Netherlands with Strict viral loads. Differences in heritability between cohorts, subdivisions and methods for estimation carry implications for the biology of heritability which offer interesting avenues for future modeling work. Experimental and epidemiological research are also required to directly identify viral factors which contribute to variance in SPVL, as well as exploring the impact of treatment on virulence evolution.

6 Acknowledgements

For advice, discussions and technical help the authors would like to thank Luuk Gras, Frank de Wolf, Neil Ferguson, Andrew Rambaut, Simon Cauchemez, Lulla Opatowski, Oliver Rattman, Thibaut Jombart, Deirdre Hollingsworth, Federico Calboli, Jeff Eaton, Matthew Hartfield, Melissa Ward, Emma Hodcroft, Andrew Leigh Brown, Joshua Herbeck, Tanja Stadler, Roland Regoes and Sebastian Bonhoeffer. We also thank anonymous reviewers for their helpful comments which have helped strengthen this work.

6.1 Swiss HIV Cohort Study

The SHCS was supported by the Swiss National Science Foundation (SNF grant #33CS30-134277). Further support was provided by, SNF grant #324730-130865 (to HFG) and the SHCS projects #470, 528, 569, 683, the SHCS Research Foundation, the Jacob-Stiftung, Zürich (to HFG), by an unrestricted research grant of the Union Bank of Switzerland, in the name of an anonymous donor to HFG, and an unrestricted research from Gilead, Switzerland (to HFG), and by the University of Zurichs Clinical research Priority Program (CRPP) "Viral infectious diseases: Zurich Primary HIV Infection Study" (to HFG). The funders had no role in study design, data collection and analysis, decision to publish, or preparation of the manuscript.

We thank the patients who participate in the SHCS; the physicians and study nurses for excellent patient care; the resistance laboratories for high quality genotypic drug resistance testing; SmartGene, Zug, Switzerland for technical support; Brigitte Remy, Martin Rickenbach, MD, F. Schöni-Affolter and Yannick Vallet from the SHCS Data Center in Lausanne for the data management and Danièle Perraudin and Mirijam Minichiello for administrative assistance. The members of the Swiss HIV Cohort Study are: Aubert V, Barth J, Battegay M, Bernasconi E, Böni J, Bucher HC, Burton-Jeangros C, Calmy A, Cavassini M, Egger M, Elzi L, Fehr J, Fellay J, Francioli P, Furrer H (Chairman of the Clinical and Laboratory Committee), Fux CA, Gorgievski M, Günthard H (President of the SHCS), Haerry D (deputy of "Positive Council"), Hasse B, Hirsch HH, Hirschel B, Hösli I, Kahlert C, Kaiser L, Keiser O, Kind C, Klimkait T, Kovari H, Ledergerber B, Martinetti G, Martinez de Tejada B, Metzner K, Mller N, Nadal D, Pantaleo G, Rauch A (Chairman of the Scientific

Board), Regenass S, Rickenbach M (Head of Data Center), Rudin C (Chairman of the Mother and Child Substudy), Schmid P, Schultze D, Schöni-Affolter F, Schüpbach J, Speck R, Taffé P, Tarr P, Telenti A, Trkola A, Vernazza P, Weber R, Yerly S.

6.2 Rakai Health Sciences Programme

The study was funded in part by the Department of Intramural Research, National Institute of Allergy and Infectious Diseases [NIAID], National Institutes of Health [NIH](to OL). We thank the participants and the study team of the Rakai Community Cohort Study, which was supported by (i) the Bill and Melinda Gates Foundation (22006.03), (ii) the National Institutes of Health (NIH), Division of Allergy and Infectious Diseases (U1AI51171 and 1UO1AI075115-O1A1), (iii) the Department of the Army, U.S. Army Medical Research and Materiel Command Cooperative Agreement (DAMD17-98-2-8007), and (v) the Henry M. Jackson Foundation (5D43TW00010). We thank the participants of the Rakai Health Sciences Program and the physicians, nurses, data managers and laboratory specialists who provide such excellent care.

The members of the Rakai Health Sciences Program are: Sewankambo N, Serwadda D, Kigozi G, Nalugoda F, Wabwire-Mangen F, Musoke R, Kagaayi J, Ssekubugu R, Lutalo T, Kiddugavu M, Makumbi F, Ssempijja V, Galiwango RM, Nantume B, Kiwanuka N, Boaz I, Kong X, Quinn TC, Reynolds SJ, Wawer MJ, Gray RH.

6.3 ATHENA National Observational Cohort, Netherlands

This is a collaboration between the following treatment centres and individuals in the Netherlands. Director: Prof Dr. F. de Wolf. Data analysis group: Dr. D.O. Bezemer, Drs. L.A.J. Gras, Dr. A.M. Kesselring, Dr. A.I. van Sighem, Dr. Ir. C. Smit. Data collection: Drs. S. Zaheri. Participating centres (site coordinating physicians): Academisch Medisch Centrum bij de Universiteit van Amsterdam, Amsterdam: Prof. dr. J.M. Prins, Prof. dr. T.W. Kuijpers, Dr. H.J. Scherpbier, Dr. J.T.M. van der Meer, Dr. F.W.M.N. Wit, Dr. M.H. Godfried, Prof. dr. P. Reiss, Prof. dr. T. van der Poll, Dr. F.J.B. Nellen, Prof. dr. J.M.A. Lange, Dr. S.E. Geerlings, Dr. M. van Vugt, Drs. D. Pajkrt, Drs. J.C. Bos, Drs. M. van der Valk, Drs. M.L. Grijsen, Dr. W.J. Wiersinga. Academisch Ziekenhuis Maastricht, Maastricht: Dr. S. Lowe, Dr. G. Schreij, Dr. A. Oude Lashof, Dr. D. Posthouwer. Catharina-ziekenhuis, Eindhoven: Drs. M.J.H. Pronk, Dr. B. Bravenboer. Erasmus Medisch Centrum, Rotterdam: Dr. M.E. van der Ende, Dr. T.E.M.S. de Vries-Sluijs, Dr. C.A.M. Schurink, Dr. J.L. Nouwen, Dr. M.H. Nispen tot Pannerden, Dr. A. Verbon, Drs. B.J.A. Rijnders, Dr. E.C.M. van Gorp, Dr. R.J. Hassing, Drs. A.W.M. Smeulders. Erasmus Medisch CentrumSophia, Rotterdam: Dr. N.G. Hartwig, Dr. G.J.A. Driessen. Flevoziekenhuis. Almere: Dr. J. Branger. HagaZiekenhuis, Den Haag: Dr. E.F. Schippers, Dr. C. van Nieuwkoop, Drs. E.P. van Elzakker. Isala Klinieken, Zwolle: Dr. P.H.P. Groeneveld, Dr. M.A. Alleman, Drs. J.W. Bouwhuis. Kennemer Gasthuis: Dr. R. Soetekouw, Prof. dr. R.W. ten Kate. Leids Universitair Medisch Centrum, Leiden: Dr. F.P. Kroon, Prof. dr. J.T. van Dissel, Dr. S.M. Arend, Dr. M.G.J. de Boer, Drs. H. Jolink, Dr. H.J.M. ter Vollaard, Drs. M.P. Bauer. Maasstadziekenhuis, Rotterdam: Dr. J.G. den Hollander, Dr. K. Pogany. Medisch Centrum Alkmaar, Alkmaar: Drs. G. van Twillert, Drs. W. Kortmann. Medisch Centrum Haaglanden, Den Haag: Dr. E.M.S. Leyten, Dr. L.B.S. Gelinck. Medisch Spectrum Twente, Enschede: Drs. G.J. Kootstra, Drs. C.E. Delsing. Onze Lieve Vrouwe Gasthuis, Amsterdam: Prof. dr. K. Brinkman, Dr. W.L. Blok, Dr. P.H.J. Frissen, Drs. W.E.M. Schouten, Drs. G.E.L. van den Berk. Sint Elisabeth Ziekenhuis, Tilburg: Dr. M.E.E. van Kasteren. Sint Lucas Andreas Ziekenhuis, Amsterdam: Dr. J. Veenstra, Dr. K.D. Lettinga. Slotervaartziekenhuis, Amsterdam: Dr. J.W. Mulder, Drs. S.M.E. Vrouenraets, Dr. F.N. Lauw. Stichting Medisch Centrum Jan van Goyen, Amsterdam: Drs. A. van Eeden, Dr. D.W.M. Verhagen. Universitair Medisch Centrum Groningen, Groningen: Drs. H.G. Sprenger, Drs. R. Doedens, Dr. E.H. Scholvinck, Drs. S. van Assen, Dr. W.F.W. Bierman. Universitair Medisch Centrum Sint Radboud, Nijmegen: Dr. P.P. Koopmans, Dr. M. Keuter, Dr. A.J.A.M. van der Ven, Dr. H.J.M. ter Hofstede, Dr. A.S.M. Dofferhoff, Dr. A Warris, Dr. R. van Crevel. Universitair Medisch Centrum Utrecht, Utrecht: Prof. dr. A.I.M. Hoepelman, Dr. T. Mudrikova, Dr. M.M.E. Schneider, Dr. P.M. Ellerbroek, Dr. J.J. Oosterheert, Dr. J.E. Arends, Dr. M.W.M. Wassenberg, Dr. R.E. Barth. Vrije Universiteit Amsterdam, Amsterdam: Dr. M.A. van Agtmael, Dr. R.M.

Perenboom, Drs. F.A.P. Claessen, Dr. M. Bomers, Dr. E.J.G. Peters. Wilhelmina Kinderziekenhuis, Utrecht: Dr. S.P.M. Geelen, Dr. T.F.W. Wolfs, Dr. L.J. Bont. Ziekenhuis Rijnstate, Arnhem: Dr. C. Richter, Dr. J.P. van der Berg, Dr. E.H. Gisolf. Admiraal De Ruyter Ziekenhuis, Vlissingen: Drs. M. van den Berge, Drs. A. Stegeman. Medisch Centrum Leeuwarden, Leeuwarden: Dr. M.G.A. van Vonderen, Drs. D.P.F. van Houte. Medisch Centrum Zuiderzee, Lelystad: Dr. S. Weijer, Dr. R. el Moussaoui. Sint Elisabeth Hospitaal, Willemstad - Curaçao: Dr. C. Winkel, Drs. F. Muskiet, Drs. Durand, Drs. R. Voigt.

References

[1] L. J. Abu-Raddad, P. Patnaik, and J. G. Kublin. "Dual infection with HIV and malaria fuels the spread of both diseases in sub-Saharan Africa". In: *Science* 314.5805 (2006), pp. 1603–1606.

[2] S. Alizon et al. "Phylogenetic Approach Reveals That Virus Genotype Largely Determines HIV Set-Point Viral Load". In: *Plos Pathogens* 6.9 (2010), e1001123.

[3] S. Alizon et al. "Virulence evolution and the trade-off hypothesis: history, current state of affairs and the future". In: *Journal of Evolutionary Biology* 22.2 (2009), pp. 245–259.

[4] M. C. Atkins et al. "Fluctuations of HIV load in semen of HIV positive patients with newly acquired sexually transmitted diseases". In: *British Medical Journal* 313.7053 (1996), pp. 341–342.

[5] D. Bezemer et al. "27 years of the HIV epidemic amongst men having sex with men in the Netherlands: an in depth mathematical model-based analysis." In: *Epidemics* 2.2 (June 2010), pp. 66–79. ISSN: 1878-0067. DOI: 10.1016/j.epidem.2010.04.001. URL: http://apps.webofknowledge.com/full_record.do?product=UA\&search_mode=GeneralSearch\&qid=2\&SID=R1bknf52BpBi5I9FejJ\&page=1\&doc=4.

[6] D. Bezemer et al. "Transmission networks of HIV-1 among men having sex with men in the Netherlands". In: *AIDS* 24.2 (2010), pp. 271–282.

[7] S. P. Blomberg, T. Garland, and A. R. Ives. "Testing for phylogenetic signal in comparative data: Behavioral traits are more labile". In: *Evolution* 57.4 (2003), pp. 717–745.

[8] P. Borrow et al. "Antiviral pressure exerted by HIV-1-specific cytotoxic T lymphocytes (CTLs) during primary infection demonstrated by rapid selection of CTL escape virus". In: *Nature Medicine* 3.2 (1997), pp. 205–11.

[9] M. Carrington and S. J. O'Brien. "The influence of HLA genotype on AIDS". In: *Annual Review of Medicine-Selected Topics in the Clinical Sciences* 54 (2003), pp. 535–551.

[10] U. Dieckmann et al. *Cambridge Studies in Adaptive Dynamics. Adaptive dynamics of infectious diseases: In pursuit of virulence management.* 2002. URL: http://user.iiasa.ac.at/~dieckman/reprints/BookExcerpts-DieckmannEtal2002.pdf.

[11] C. A. Donnelly et al. "Gender difference in HIV-1 RNA viral loads". In: *HIV Medicine* 6.3 (2005), pp. 170–178.

[12] G.J. Etherington, J. Dicks, and I.N. Roberts. "Recombination Analysis Tool (RAT): a program for the high-throughput detection of recombination". English. In: *BIOINFORMATICS* 21.3 (2005), 278–281. ISSN: 1367-4803. DOI: {10.1093/bioinformatics/bth500}.

[13] D.S. Falconer. *Introduction to quantitative genetics.* 1960.

[14] J. Fellay et al. "Common Genetic Variation and the Control of HIV-1 in Humans". In: *Plos Genetics* 5.12 (2009), pp. –.

[15] J. Felsenstein. "Phylogenies and Quantitative Characters". In: *Annual Review of Ecology and Systematics* 19 (1988), pp. 445–471.

[16] R. A. Fisher. "The correlation between relatives on the supposition of Mendelian inheritance". In: *Transactions of the Royal Society of Edinburgh* 52 (1918), pp. 399–433.

[17] C. Fraser et al. "Variation in HIV-1 set-point viral load: Epidemiological analysis and an evolutionary hypothesis". In: *Proceedings of the National Academy of Sciences of the United States of America* 104 (2007), pp. 17441–17446.

[18] R. P. Freckleton. "The seven deadly sins of comparative analysis". In: *Journal of evolutionary biology* 22.7 (2009). Freckleton, R P Switzerland J Evol Biol. 2009 Jul;22(7):1367-75. Epub 2009 Jun 5., pp. 1367–75.

[19] R. P. Freckleton, P. H. Harvey, and M. Pagel. "Phylogenetic analysis and comparative data: A test and review of evidence". In: *American Naturalist* 160.6 (2002), pp. 712–726.

[20] S. Gandon et al. "Imperfect vaccines and the evolution of pathogen virulence". In: *Nature* 414.6865 (2001), pp. 751–756.

[21] R. B. Geskus et al. "The HIV RNA setpoint theory revisited". In: *Retrovirology* 4 (2007), p. 65.

[22] L. Gras et al. "Viral load levels measured at set-point have risen over the last decade of the HIV epidemic in the Netherlands". In: *PLoS One* 4.10 (2009), e7365.

[23] S. Guindon and O. Gascuel. "A simple, fast, and accurate algorithm to estimate large phylogenies by maximum likelihood". In: *Systematic biology* 52.5 (2003). Guindon, Stephane Gascuel, Olivier England Syst Biol. 2003 Oct;52(5):696-704., pp. 696–704.

[24] F. M. Hecht et al. "HIV RNA level in early infection is predicted by viral load in the transmission source". In: *AIDS* 24.7 (2010), pp. 941–945.

[25] D. R. Henrard et al. "Natural History Of HIV-1 Cell-free Viremia". In: *Jama-Journal of the American Medical Association* 274.7 (1995), pp. 554–558.

[26] J. T. Herbeck et al. "Is the virulence of HIV changing? A meta-analysis of trends in prognostic markers of HIV disease progression and transmission". In: *AIDS* 26.2 (2012), pp. 193–205.

[27] D. D. Ho et al. "Rapid Turnover of Plasma Virions and Cd4 Lymphocytes in HIV-1 Infection". In: *Nature* 373.6510 (1995), pp. 123–126.

[28] E. Hodcroft et al. "Population-based Study Indicates Viral Genetic Effect on HIV Virulence Is Small but Significant". In: 2012.

[29] T. D. Hollingsworth et al. "HIV-1 Transmitting Couples Have Similar Viral Load Set-Points in Rakai, Uganda". In: *Plos Pathogens* 6.5 (2010).

[30] E. A. Housworth, E. P. Martins, and M. Lynch. "The phylogenetic mixed model". In: *American Naturalist* 163.1 (2004), pp. 84–96.

[31] J. P. Ji and L. A. Loeb. "Fidelity of HIV-1 Reverse-Transcriptase Copying a Hypervariable Region of the HIV-1 Env Gene". In: *Virology* 199.2 (1994), pp. 323–330.

[32] L. E. Jones and A. S. Perelson. "Transient viremia, plasma viral load, and reservoir replenishment in HIV-Infected patients on antiretroviral therapy". In: *Jaids-Journal of Acquired Immune Deficiency Syndromes* 45.5 (2007), pp. 483–493.

[33] E. L. Korenromp et al. "Clinical prognostic value of RNA viral load and CD4 cell counts during untreated HIV-1 infection–a quantitative review". In: *PLoS One* 4.6 (2009), e5950.

[34] R. D. Kouyos et al. "Molecular epidemiology reveals long-term changes in HIV type 1 subtype B transmission in Switzerland." In: *The Journal of infectious diseases* 201.10 (May 2010), pp. 1488–97. ISSN: 1537-6613. DOI: 10.1086/651951. URL: http://www.ncbi.nlm.nih.gov/pubmed/20384495.

[35] A. C. van der Kuyl et al. "HIV RNA levels in transmission sources only weakly predict plasma viral load in recipients". In: *AIDS* 24.10 (2010), pp. 1607–1608.

[36] B. Ledergerber et al. "The Swiss HIV Cohort Study - Rationale, Organization and Selected Base-Line Characteristics". In: *Sozial-Und Praventivmedizin* 39.6 (1994), pp. 387–394.

[37] A. J. Leslie et al. "HIV evolution: CTL escape mutation and reversion after transmission". In: *Nature Medicine* 10.3 (2004), pp. 282–289.

[38] N. Mantel. "The detection of disease clustering and a generalized regression approach". In: *Cancer Res* 27.2 (1967), pp. 209–20.

[39] J. Masel et al. "Fluctuations in HIV-1 viral load are correlated to CD4(+) T-lymphocyte count during the natural course of infection". In: *Journal of Acquired Immune Deficiency Syndromes* 23.5 (2000), pp. 375–379.

[40] J. W. Mellors et al. "Prognosis in HIV-1 infection predicted by the quantity of virus in plasma". In: *Science* 272.5265 (1996), pp. 1167–1170.

[41] V. Müller, C. Fraser, and J. T. Herbeck. "A Strong Case for Viral Genetic Factors in HIV Virulence". In: *Viruses-Basel* 3.3 (2011), pp. 204–216.

[42] V. Müller et al. "Increasing clinical virulence in two decades of the Italian HIV epidemic". In: *Plos Pathogens* 5.5 (2009), e1000454.

[43] V. Novitsky et al. "Intra-host evolutionary rates in HIV-1C env and gag during primary infection." In: *Infection, genetics and evolution : journal of molecular epidemiology and evolutionary genetics in infectious diseases* (Mar. 2013). ISSN: 1567-7257. DOI: 10.1016/j.meegid.2013.02.023. URL: http://www.ncbi.nlm.nih.gov/pubmed/23523818.

[44] M. A. Nowak et al. "Antigenic Diversity Thresholds and the Development of AIDS". In: *Science* 254.5034 (1991), pp. 963–969.

[45] E. Paradis, J. Claude, and K. Strimmer. "APE: Analyses of Phylogenetics and Evolution in R language". In: *Bioinformatics* 20.2 (2004), pp. 289–290.

[46] S. Pavoine and C. Ricotta. "Testing for phylogenetic signal in biological traits: the ubiquity of cross-product statistics". In: *Evolution* 1967 (Oct. 2012), no–no. ISSN: 00143820. DOI: `10.1111/j.1558-5646.2012.01823.x`. URL: `http://doi.wiley.com/10.1111/j.1558-5646.2012.01823.x`.

[47] S. Pavoine et al. "Testing for phylogenetic signal in phenotypic traits: New matrices of phylogenetic proximities". In: *Theoretical Population Biology* 73.1 (2008), pp. 79–91.

[48] A. F. Y. Poon et al. "Adaptation to Human Populations Is Revealed by Within-Host Polymorphisms in HIV-1 and Hepatitis C Virus". In: *PLoS Pathog* 3.3 (Mar. 2007), e45. DOI: `10.1371/journal.ppat.0030045`.

[49] D. Posada and K. A. Crandall. "MODELTEST: testing the model of DNA substitution". In: *Bioinformatics* 14.9 (1998), pp. 817–818.

[50] B. A. Richardson et al. "Comparison of human immunodeficiency virus type 1 viral loads in Kenyan women, men, and infants during primary and early infection". In: *Journal of Virology* 77.12 (2003), pp. 7120–7123.

[51] D. Richman et al. "Human-Immunodeficiency-Virus Type-1 Mutants Resistant to Nonnucleoside Inhibitors of Reverse-Transcriptase Arise in Tissue-Culture". In: *Proceedings of the National Academy of Sciences of the United States of America* 88.24 (1991), pp. 11241–11245.

[52] P. Rieder et al. "HIV-1 transmission after cessation of early antiretroviral therapy among men having sex with men." In: *AIDS (London, England)* 24.8 (May 2010), pp. 1177–83. ISSN: 1473-5571. DOI: `10.1097/QAD.0b013e328338e4de`. URL: `http://www.ncbi.nlm.nih.gov/pubmed/20386427`.

[53] Alicia Sanchez-Mazas. "African Diversity From the HLA Point of View : Influence of Genetic Drift , Geography , Linguistics , and Natural Selection". In: 62 (2001), pp. 937–948.

[54] F. Schoeni-Affolter et al. "Cohort Profile: The Swiss HIV Cohort Study". In: *International Journal of Epidemiology* 39.5 (2010), pp. 1179–1189.

[55] R. Schuurman et al. "Rapid Changes in Human-Immunodeficiency-Virus Type-1 Rna Load and Appearance of Drug-Resistant Virus Populations in Persons Treated with Lamivudine (3tc)". In: *Journal of Infectious Diseases* 171.6 (1995), pp. 1411–1419.

[56] G. Shirreff et al. "Transmission Selects for HIV-1 Strains of Intermediate Virulence: A Modelling Approach". In: *Plos Computational Biology* 7.10 (2011).

[57] A. Stamatakis. "RAxML-VI-HPC: Maximum likelihood-based phylogenetic analyses with thousands of taxa and mixed models". In: *Bioinformatics* 22.21 (2006), pp. 2688–2690.

[58] K. Sundberg et al. "Incomparability of results between phylogenetic search programs". In: (2008), pp. 81–4. URL: `http://www.ncbi.nlm.nih.gov/CBBresearch/Fellows/Carroll/pubs/sundberg2008incomparability.pdf`.

[59] J. M. Tang et al. "HLA allele sharing and HIV type 1 viremia in seroconverting Zambians with known transmitting partners". In: *AIDS Research and Human Retroviruses* 20.1 (2004), pp. 19–25.

[60] S. Tavaré. "Some Probabilistic and Statistical Problems in the Analysis of DNA Sequences". In: *American Mathematical Society: Lectures on Mathematics in the Life Sciences.* Vol. 17. Amer Mathematical Society, 1986, pp. 57–86. ISBN: 0821811673. URL: `http://www.amazon.com/exec/obidos/redirect?tag=citeulike07-20\&path=ASIN/0821811673`.

[61] R Development Core Team. *R: A language and environment for statistical computing.* 2011.

[62] L. S. Uhlenbeck G.E.; Ornstein. "On the Theory of the Brownian Motion". In: *Phys. Rev.* 36 (1930), 823841.

[63] UNAIDS. *HIV and AIDS Estimates.* 2009. URL: `http://www.unaids.org/en/regionscountries/countries/`.

[64] M. J. Wawer et al. "A randomized, community trial of intensive sexually transmitted disease control for AIDS prevention, Rakai, Uganda". In: *AIDS* 12.10 (1998), pp. 1211–1225.

[65] M. J. Wawer et al. "Control of sexually transmitted diseases for AIDS prevention in Uganda: a randomised community trial". In: *Lancet* 353.9152 (1999), pp. 525–535.

[66] X. Wei et al. "Viral dynamics in human immunodeficiency virus type 1 infection". In: *Nature* 373.6510 (1995), pp. 117–22.

[67] F. de Wolf et al. “AIDS prognosis based on HIV-1 RNA, CD4+ T-cell count and function: Markers with reciprocal predictive value over time after seroconversion”. In: *AIDS* 11.15 (1997), pp. 1799–1806.

[68] T. F. W. Wolfs et al. “Naturally-Occurring Mutations within HIV-1 V3 Genomic Rna Lead to Antigenic Variation Dependent on a Single Amino-Acid Substitution”. In: *Virology* 185.1 (1991), pp. 195–205.

[69] V. von Wyl et al. “Emergence of HIV-1 drug resistance in previously untreated patients initiating combination antiretroviral treatment: a comparison of different regimen types.” In: *Archives of internal medicine* 167.16 (Sept. 2007), pp. 1782–90. ISSN: 0003-9926. DOI: 10.1001/archinte.167.16.1782. URL: http://www.ncbi.nlm.nih.gov/pubmed/17846398.

[70] Z. H. Yang. “Among-site rate variation and its impact on phylogenetic analyses”. In: *Trends in Ecology and Evolution* 11.9 (1996), pp. 367–372.

[71] S. Yerly et al. “Transmission of antiretroviral-drug-resistant HIV-1 variants”. In: *Lancet* 354.9180 (1999), pp. 729–733.
