# Supplementary material for: How effectively can HIV phylogenies be used to measure heritability?
Source: Evol Med Public Health. 2013 Sep 13;2013(1):209–24. doi: 10.1093/emph/eot019 (PMC3850537; doi:10.1093/emph/eot019)
Supplement: Supplementary Data [file supp_eot019_suppl_data.zip › Latex_bits.tar.gz!/Latex_bits/SI_phylo_swiss.pdf]

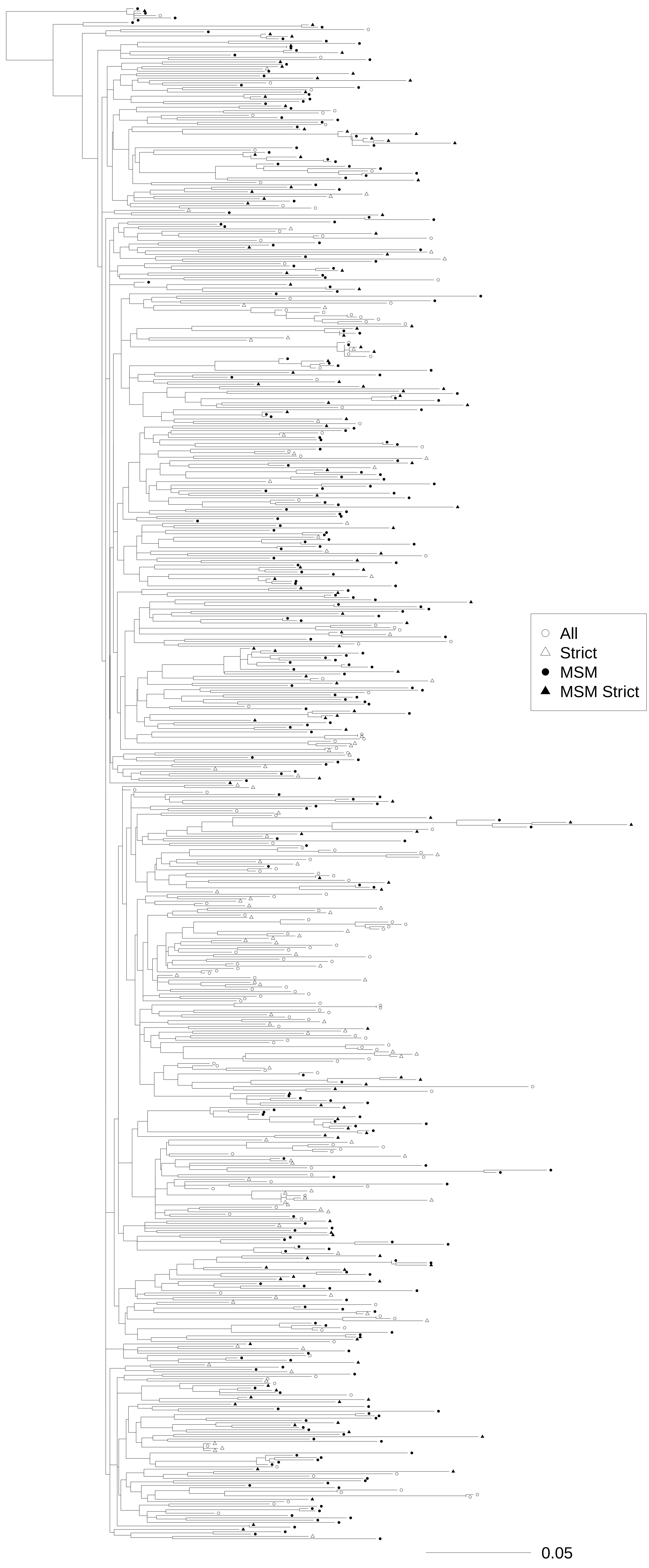

All
Strict
MSM
MSM Strict
0.05
