# Supplementary material for: How effectively can HIV phylogenies be used to measure heritability?
Source: Evol Med Public Health. 2013 Sep 13;2013(1):209–24. doi: 10.1093/emph/eot019 (PMC3850537; doi:10.1093/emph/eot019)
Supplement: Supplementary Data [file supp_eot019_suppl_data.zip › Latex_bits.tar.gz!/Latex_bits/SI_rakai_p24_eps_1_herithist_h2.pdf]

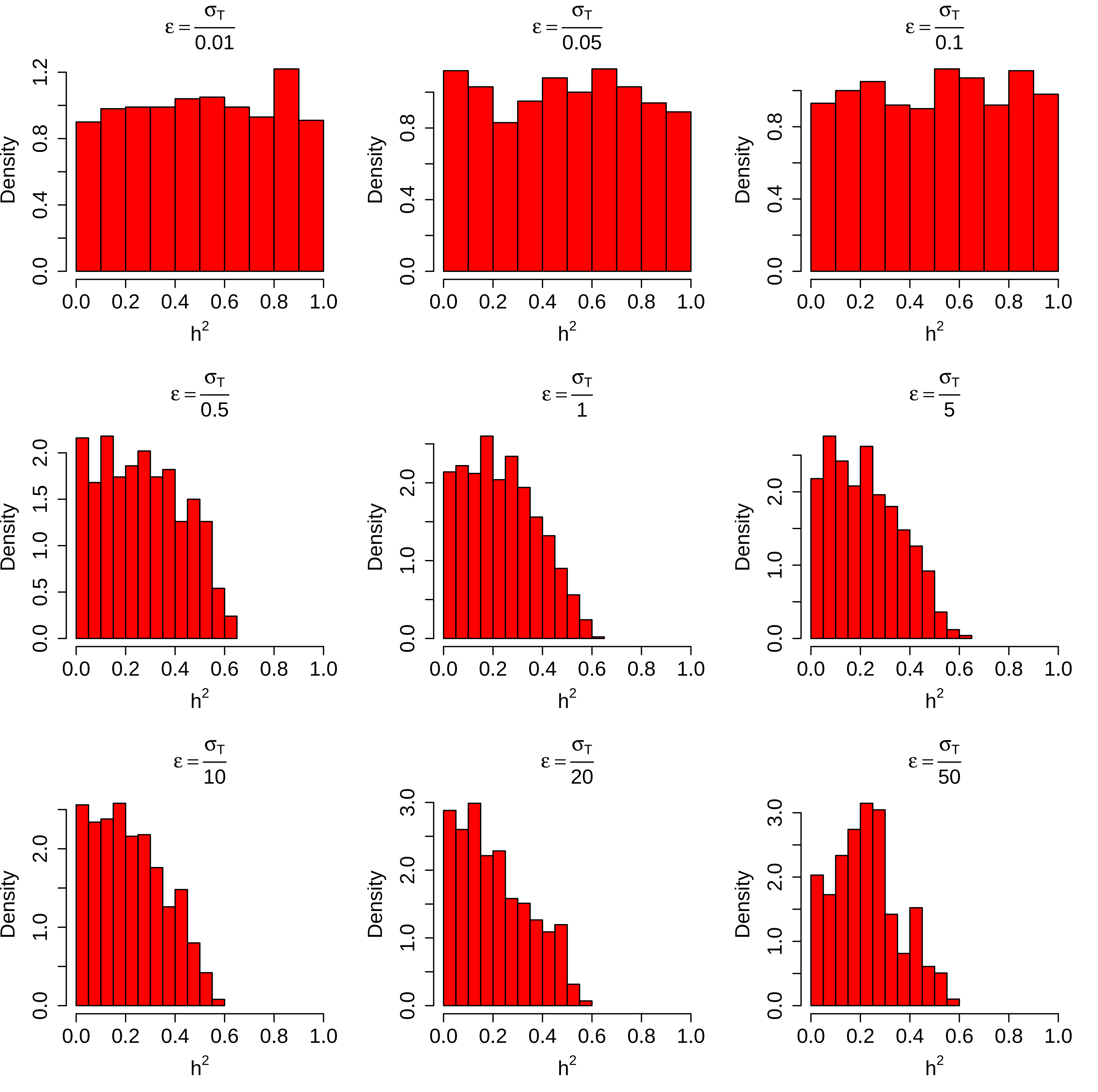

$\varepsilon = \frac{\sigma_T}{0.01}$
$\varepsilon = \frac{\sigma_T}{0.05}$
$\varepsilon = \frac{\sigma_T}{0.1}$
$\varepsilon = \frac{\sigma_T}{0.5}$
$\varepsilon = \frac{\sigma_T}{1}$
$\varepsilon = \frac{\sigma_T}{5}$
$\varepsilon = \frac{\sigma_T}{10}$
$\varepsilon = \frac{\sigma_T}{20}$
$\varepsilon = \frac{\sigma_T}{50}$
Density
h^2
