# Supplementary material for: How effectively can HIV phylogenies be used to measure heritability?
Source: Evol Med Public Health. 2013 Sep 13;2013(1):209–24. doi: 10.1093/emph/eot019 (PMC3850537; doi:10.1093/emph/eot019)
Supplement: Supplementary Data [file supp_eot019_suppl_data.zip › Latex_bits.tar.gz!/Latex_bits/SI_validate_simrand_swiss_MSM_strict_orig_1011121314_pvalue_herit_h2.pdf]

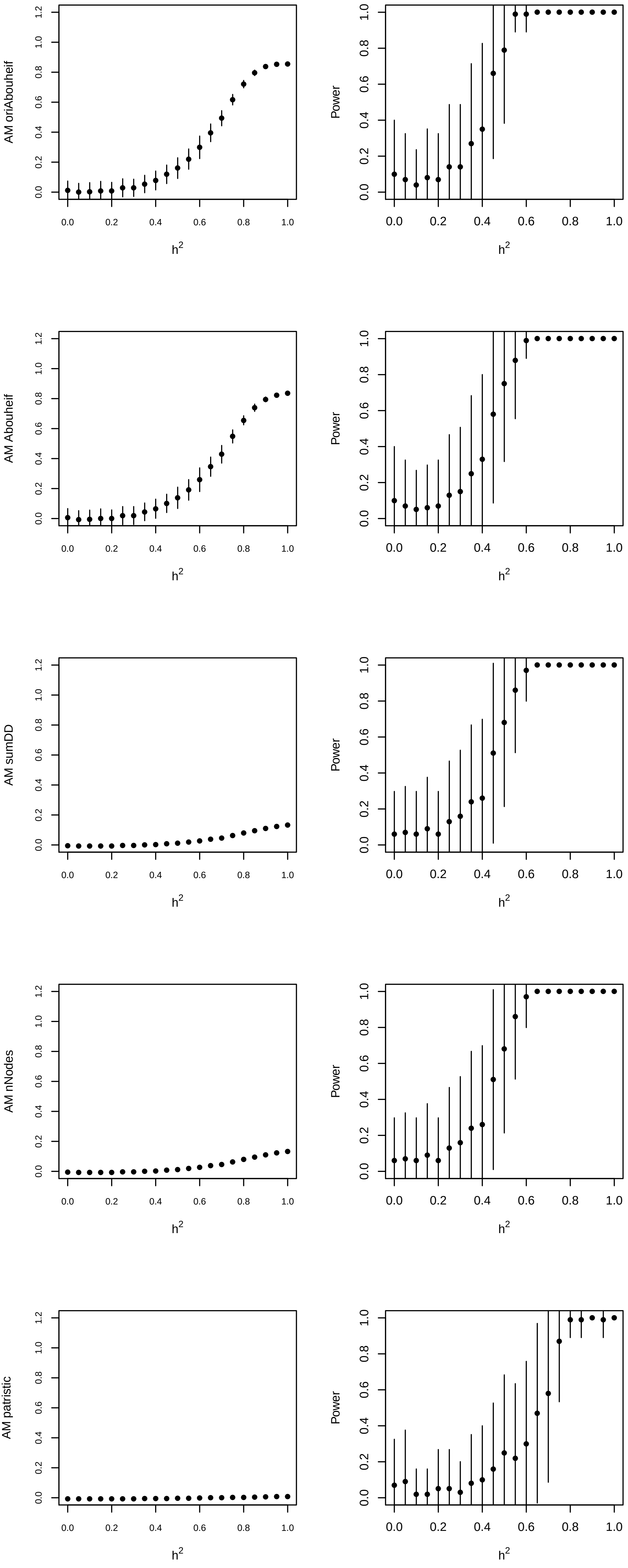

AM oriAbouheif
Power
AM Abouheif
Power
AM sumDD
Power
AM nNodes
Power
AM patristic
Power
h2
