# Supplementary material for: How effectively can HIV phylogenies be used to measure heritability?
Source: Evol Med Public Health. 2013 Sep 13;2013(1):209–24. doi: 10.1093/emph/eot019 (PMC3850537; doi:10.1093/emph/eot019)
Supplement: Supplementary Data [file supp_eot019_suppl_data.zip › Latex_bits.tar.gz!/Latex_bits/SI_validate_simrand_swiss_MSM_strict_orig_2345_pvalue_herit_h2.pdf]

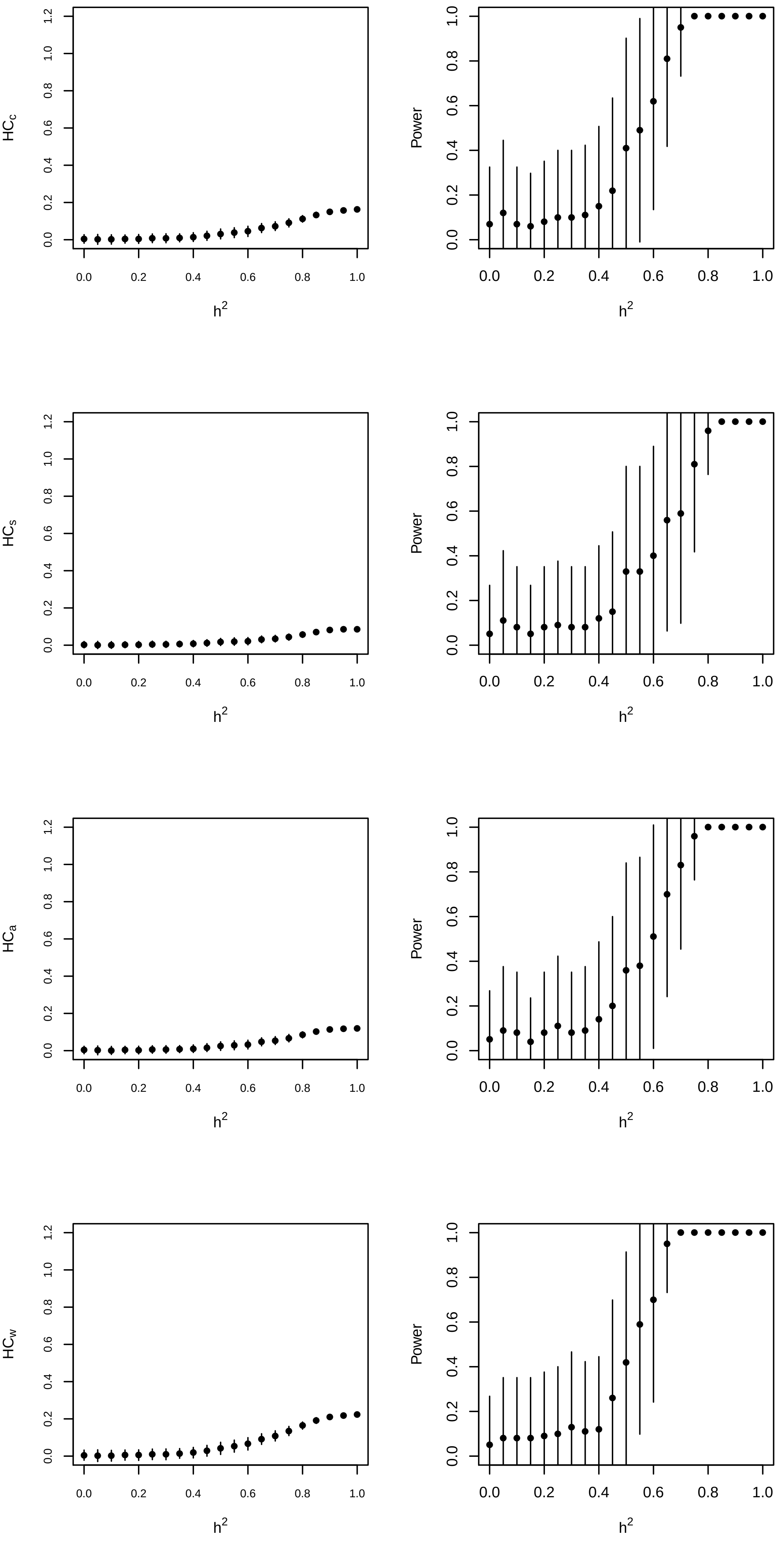
HCc
Power
h2
HCs
Power
h2
HCa
Power
h2
HCw
Power
h2
0.0
0.2
0.4
0.6
0.8
1.0
1.2
