# Supplementary material for: How effectively can HIV phylogenies be used to measure heritability?
Source: Evol Med Public Health. 2013 Sep 13;2013(1):209–24. doi: 10.1093/emph/eot019 (PMC3850537; doi:10.1093/emph/eot019)
Supplement: Supplementary Data [file supp_eot019_suppl_data.zip › Latex_bits.tar.gz!/Latex_bits/swiss_19_herithist_h2.pdf]

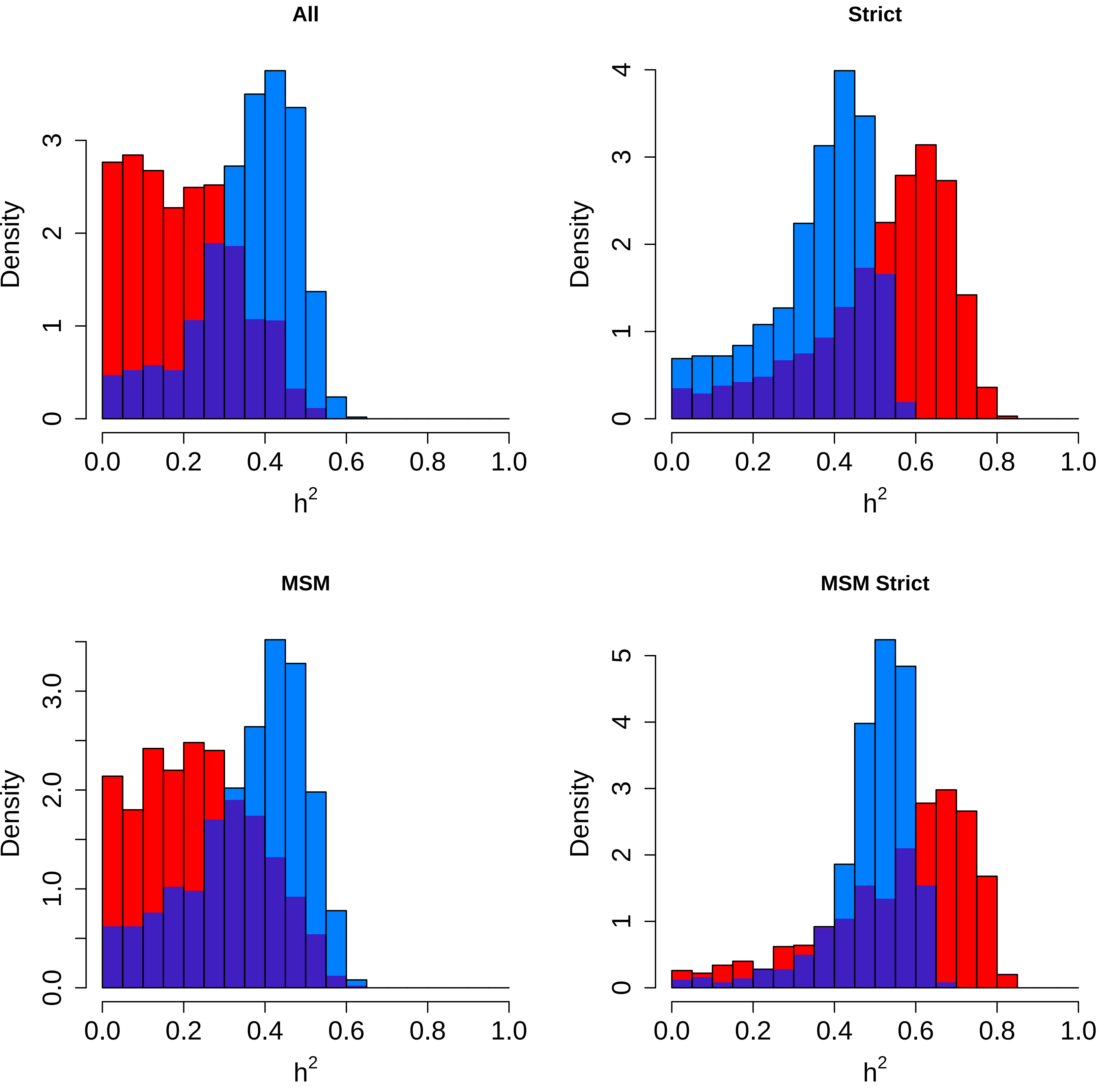
All
Strict
MSM
MSM Strict
Density
h^2
0.0
0.2
0.4
0.6
0.8
1.0
0
1
2
3
4
5
0.0
1.0
2.0
3.0
