# Supplementary material for: How effectively can HIV phylogenies be used to measure heritability?
Source: Evol Med Public Health. 2013 Sep 13;2013(1):209–24. doi: 10.1093/emph/eot019 (PMC3850537; doi:10.1093/emph/eot019)
Supplement: Supplementary Data [file supp_eot019_suppl_data.zip › Latex_bits.tar.gz!/Latex_bits/validate_simrand_swiss_all_orig_1567911_pvalue_herit_h2.pdf]

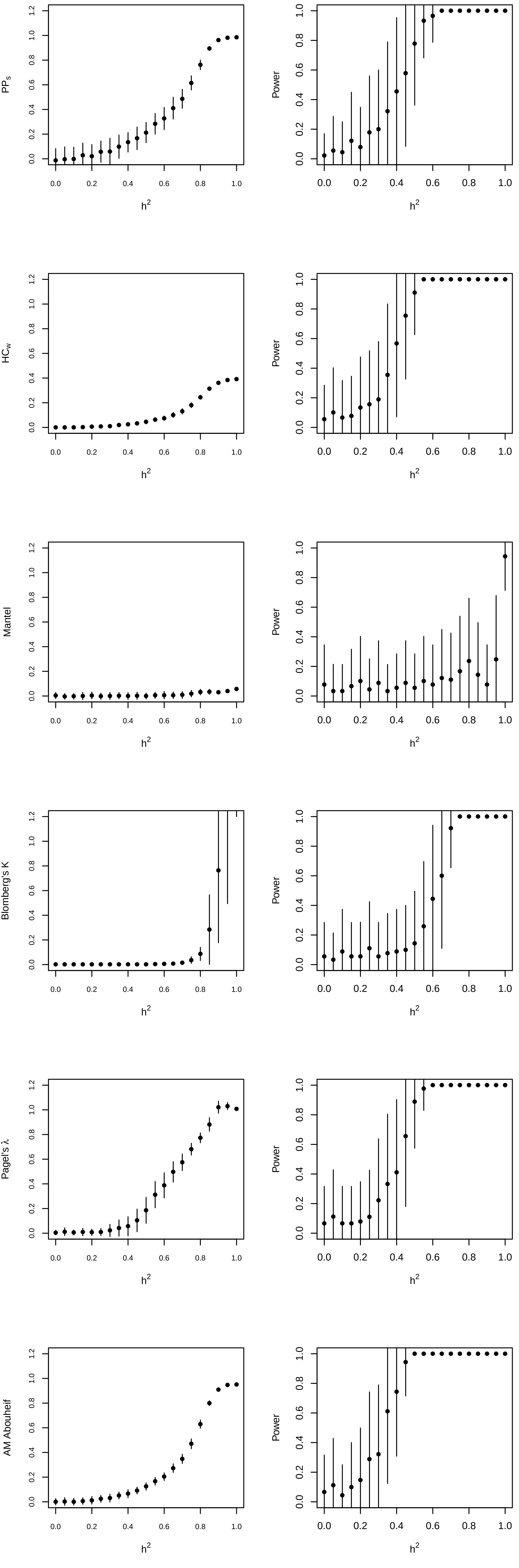
PP$_s$
Power
h^2
HC$_w$
Mantel
Blomberg's K
Pagel's λ
AM Abouheif
0.0
0.2
0.4
0.6
0.8
1.0
1.2
